# RIPK3 Inhibition Mitigates Denervated Muscle Atrophy via NOX4‐Mediated Mitochondrial Restoration and Inflammation Suppression

**DOI:** 10.1002/jcsm.70311

**Published:** 2026-05-01

**Authors:** Yuntian Shen, Chen Zhang, Zihao Zhao, Jia Yi, Xinlei Yao, Bingqian Chen, Hualin Sun

**Affiliations:** ^1^ Key Laboratory of Neuroregeneration of Jiangsu and Ministry of Education Medical School of Nantong University, Co‐Innovation Center of Neuroregeneration, Nantong University Nantong Jiangsu China; ^2^ Department of Orthopedics Changshu Hospital Affiliated to Soochow University, First People's Hospital of Changshu City Changshu Jiangsu China

**Keywords:** denervation, mitochondrial dysfunction, muscle atrophy, NOX4, RIPK3

## Abstract

**Background:**

Peripheral nerve injury‐induced muscle atrophy shares core pathophysiological features with systemic wasting disorders including cachexia and sarcopenia, yet early molecular triggers remain undefined. This study investigates the pathogenic role of receptor‐interacting protein kinase 3 (RIPK3) in denervation atrophy.

**Methods:**

Sciatic denervation was induced in rats for initial time‐course transcriptomics and in mice for genetic and pharmacological studies. Assessments in wild‐type and RIPK3‐knockout mice included transcriptomics (RNA‐seq, qPCR), muscle morphology (wet weight ratio, cross‐sectional area), histological inflammation (H&E, CD68 immunofluorescence), mitochondrial function (complex I/V activity, ultrastructure and biogenesis/fission regulators), STRING analysis to identify downstream effectors, validated key effectors NOX2 and NOX4 (qPCR/Western blotting) and associated redox status (DHE staining), and analysis of myofibrillar protein content and proteolytic markers (Western blotting). Confirmatory studies included RIPK3 overexpression in C2C12 myotubes and its pharmacological inhibition (GSK872) in mice.

**Results:**

RIPK3 emerged from transcriptomic analysis as an early upregulated mediator in denervated muscle, with protein levels increasing approximately threefold at 36 h post‐injury. Genetic ablation of RIPK3 attenuated muscle atrophy, as shown by improved gastrocnemius wet weight ratio (*p* = 0.0110). This protective effect was directly evidenced by a 40.7% increase in cross‐sectional area (*p* = 0.04). The morphological preservation was accompanied by markedly suppressed expression of key atrophy markers, including MAFbx, MuRF1 and FoxO3a (all *p* < 0.01), and preserved MHC levels (*p* = 0.0278). Mechanistically, RIPK3 knockout reduced inflammation, enhanced oxidative phosphorylation (GSEA FDR < 0.001) and partially restored mitochondrial function, evidenced by significantly increased complex I (*p* = 0.0438) and complex V (*p* < 0.001) activity, preserved ultrastructure, upregulated PGC‐1α and NRF2 (both *p* < 0.05) and downregulated mitochondrial fission proteins (p‐DRP1, MFF, FIS1; all *p* < 0.01). STRING analysis predicted NOX4 as a key downstream effector, validated by reduced NOX4 protein (−46.6%, *p* = 0.0366) and a consequent 52.2% decrease in ROS accumulation (*p* < 0.001). Consistently, RIPK3 overexpression in C2C12 myotubes elevated NOX4 (*p* = 0.0046) and atrophy markers, whereas pharmacological inhibition of RIPK3 in mice replicated the protective phenotype, increasing muscle wet weight ratio (*p* = 0.0277) and suppressing NOX4 (*p* = 0.0398) and proteolytic markers.

**Conclusions:**

Denervation activates RIPK3 as a master regulator that drives muscle atrophy via NOX4/ROS‐induced mitochondrial dysfunction, sustained inflammation and ubiquitin–proteasome activation. Targeting RIPK3 preserves muscle mass and may offer a novel therapeutic strategy for neurogenic muscle atrophy, with possible implications for related wasting disorders.

## 1 Introduction

Skeletal muscle, constituting approximately 40% of total body mass, serves as the primary metabolic engine for energy expenditure and mechanical performance, while also functioning as an endocrine organ that regulates systemic homeostasis through myokine secretion [1, 2, 3]. Its proteostatic balance is maintained by a delicate equilibrium between mTOR‐mediated anabolism and ubiquitin–proteasome/autophagy–lysosome catabolism, which can be disrupted by denervation, immobilization, inflammation or aging, leading to net proteolysis and irreversible atrophy [4, 5]. Notably, denervation‐induced atrophy shares core pathophysiological features, including accelerated proteolysis, mitochondrial dysfunction and chronic low‐grade inflammation, with systemic wasting disorders such as cachexia and sarcopenia [6, 7]. This common pathogenic basis is reflected in the consistent upregulation of shared molecular pathways, including FoxO3/MuRF1/MAFbx‐mediated ubiquitin–proteasome activation, mitochondrial oxidative stress and NF‐κB‐driven inflammation [8, 9, 10]. Given this shared pathogenic basis, denervation models offer a tractable experimental system to identify upstream regulators that coordinate these degenerative pathways. Elucidating such regulators in denervated muscle may therefore reveal mechanistic nodes with potential relevance to multiple wasting conditions.

Among various atrophy etiologies, neurogenic muscle wasting represents the most clinically recalcitrant form, with peripheral nerve injuries alone causing over 1 million new cases annually [6, 11]. Current standard‐of‐care interventions (microsurgical nerve repair combined with immunosuppressive regimens) achieve only partial functional recovery, leaving a substantial proportion of patients with permanent disability due to denervation‐induced myofiber loss and fibrotic replacement [12, 13]. This therapeutic inadequacy stems from insufficient understanding of early molecular events driving atrophy progression and the lack of pharmacologic agents targeting upstream regulatory nodes. Our previous microarray analysis delineated a conserved pathogenic trajectory of denervation‐induced atrophy across four sequential stages: oxidative priming, inflammatory amplification, proteolytic activation and fibrotic transition [14]. Mechanistically, oxidative‐inflammatory coupling drives initial degeneration, where mitochondrial‐derived ROS not only induces biomolecular oxidation but also propagates inflammatory cascades through the activation of MAPK, NF‐κB and NLRP3 signalling pathways [15, 16]. Reciprocally, cytokine surges (TNFα, IL‐1β, IL‐6) exacerbate mitochondrial dysfunction via membrane depolarization and cristae remodelling, establishing a self‐reinforcing degenerative loop [9, 15]. Across preclinical models including aging and cancer cachexia, these inflammatory mediators orchestrate proteostatic imbalance by suppressing mTORC1‐mediated anabolism while enhancing ubiquitin–proteasome activity via JAK/STAT pathway hyperactivation [9, 17]. The development of targeted therapeutic strategies for the above‐mentioned cascade reactions has become a key path to break through the current clinical predicament.

Pharmacological interventions targeting inflammation (NSAID‐mediated COX‐2 inhibition) and proteostasis (β_2_‐adrenergic agonists) demonstrate partial efficacy in preclinical models [18, 19]. Central to its anti‐atrophic action, celecoxib primarily attenuates denervation‐induced inflammation through potent COX‐2/PGE2 axis blockade, which secondarily suppresses MuRF1/atrogin‐1 upregulation. This anti‐inflammatory core synergizes with complementary PDK1‐TRAF4‐AKT fibrosis inhibition and Prokr1‐mediated regeneration programmes to comprehensively preserve muscle mass and function [18, 20, 21]. Clinical translation confronts two interrelated challenges consisting of cardiovascular safety constraints associated with prolonged pharmacological exposure and insufficient therapeutic intervention targeting essential pathophysiological cascades. Contemporary therapeutic innovations emphasize multi‐modal interventions that coordinately regulate mitochondrial energetics, redox homeostasis and immunoinflammatory crosstalk, three mechanistically interlinked processes that synergistically activate proteolytic cascades [8, 22, 23]. This shift in pharmacological interventions has redirected research focus towards key node molecules that can simultaneously regulate multiple pathways.

Receptor‐interacting protein kinase 3 (RIPK3) emerges as a multifunctional regulator coordinating diverse pathological networks [24]. Structural bifurcation defines its dual mechanisms: The N‐terminal kinase domain orchestrates signalling transduction through autophosphorylation cascades, whereas the C‐terminal RHIM domain mediates necrosome assembly with RIPK1/MLKL to execute programmed necrosis [24]. Pharmacologically relevant non‐canonical functions include RAGE/CaMKII signalling stimulation and NLRP3 inflammasome activation, mechanisms mediating inflammatory cell death in cardiac pathologies [25, 26]. Experimental evidence reveals RIPK3's mitochondrial toxicity in diabetic nephropathy, inducing mitochondrial fragmentation, ROS accumulation and metabolic dysfunction via the MLKL‐PGAM5‐DRP1 pathway, contributing to podocyte injury [27]. Genetic ablation studies in dystrophic mice demonstrate RIPK3’s functional necessity for myocyte necrosis, with knockout models exhibiting preserved cardiorespiratory function [28, 29]. These multimodal actions position RIPK3 as a putative critical regulator integrating inflammatory, redox and bioenergetic pathways in denervation‐induced atrophy.

This study aims to investigate the pathological role of RIPK3 in denervation‐induced muscle atrophy using RIPK3 knockout murine models. Through integration of RNA sequencing (RNA‐seq)‐based transcriptomic profiling with molecular validation, we will delineate the mechanisms underlying RIPK3‐mediated regulation of atrophy progression. Additionally, the therapeutic potential of pharmacological RIPK3 inhibition will be evaluated for mitigating denervation‐induced muscle wasting. The anticipated outcomes will reveal novel molecular regulators in neurogenic atrophy and elucidate the crosstalk among mitochondrial dysfunction, oxidative stress and inflammatory signalling. These findings are expected to establish a mechanistic foundation for developing targeted therapies against muscle atrophy disorders.

## 2 Materials and Methods

### 2.1 Animals

Adult male Sprague–Dawley (SD) rats (∼200 g; Animal Laboratory of Nantong University) and adult male C57BL/6J mice (Animal Laboratory of Nantong University) weighing 24 ± 2 g were used in this study. RIPK3‐knockout (RIPK3‐KO) mice (Shanghai Southern Model Organisms Center, NM‐KO‐200619) on a C57BL/6J background were employed for genetic studies. All animals were housed under controlled environmental conditions (20°C ± 2°C, 12‐h light/dark cycle) with ad libitum access to food and water.

Rats were utilized for the initial time‐course transcriptomic analysis, whereas mice were used for all genetic and pharmacological intervention studies. For rats, a dense series of time points (0, 15 min, 30 min, 1 h, 3 h, 6 h, 12 h, 24 h, 36 h, 3 days, 7 days, 14 days, 21 days and 28 days post‐denervation) was employed to comprehensively capture the dynamic transcriptional landscape, following the extended temporal framework established in our previous study [14]. For mice, key intervals were selected within this established progression. The left sciatic nerve denervation model was established in both species under appropriate anaesthesia (ketamine/xylazine for rats; tribromoethanol for mice), involving excision of a 10‐mm nerve segment. Sham‐operated controls underwent identical surgical exposure without nerve transection, and perioperative analgesia was provided via subcutaneous buprenorphine (0.2 mg/kg). For pharmacological inhibition, the RIPK3 inhibitor GSK872 (HY‐101872, MedChemExpress, USA) was dissolved in DMSO, diluted in saline (10 mg/kg) and administered via daily intraperitoneal injection for 14 days post‐surgery. Terminal tissue collection was performed by cervical dislocation at predefined intervals. The tibialis anterior (TA) and gastrocnemius (GAS) muscles were dissected for gravimetric analysis, with the wet weight ratio (ipsilateral/contralateral) calculated. Processed specimens were cryopreserved at −80°C or chemically fixed for downstream evaluations. All procedures were approved by the Jiangsu Provincial Laboratory Animal Management Committee (Approval No. S20230409‐006 for rat studies and No. S20230709‐006 for mouse studies) and conducted in accordance with Nantong University Animal Care Guidelines.

### 2.2 Transcriptome Analysis

Total RNA was extracted from rat TA muscles collected at multiple timepoints, wild‐type mouse GAS muscles (contralateral controls and 14‐day denervated) and RIPK3‐KO mouse GAS muscles subjected exclusively to 14‐day denervation, using TRIzol Reagent (Invitrogen). RNA integrity was verified through NanoDrop 2000 spectrophotometry (Thermo Scientific) and Agilent 2100 Bioanalyzer assessment. Libraries were constructed using the VAHTS Universal V10 RNA‐seq Kit (Vazyme, China) and sequenced on an Illumina NovaSeq 6000 platform (OE Biotech, Shanghai) to generate 150‐bp paired‐end reads. Raw reads were first subjected to quality filtering using fastp v0.23.2 (minimum length: 150 bp) before alignment to the GRCm38.p6 reference genome via HISAT2. Gene expression levels were quantified using both StringTie‐derived FPKM values and HTSeq‐count. Principal component analysis (PCA) was conducted in R to assess sample clustering and biological reproducibility. Differential expression analysis was carried out using DESeq2 with thresholds of |log2FC| > 1 and *q* < 0.05. Functional enrichment analysis was performed with clusterProfiler, covering GO terms, KEGG pathways and GSEA‐validated gene sets, and results were visualized using custom ggplot2 plots. Protein interaction networks were topologically analysed via CytoHubba in Cytoscape to delineate RIPK3‐associated regulatory modules [30]. The sequencing data have been deposited in the NCBI Sequence Read Archive (Accession Number: PRJNA1302099).

### 2.3 Immunofluorescent Staining

Fresh GAS muscles were fixed in 4% paraformaldehyde (PFA, Servicebio, China) for 14–16 h at 4°C followed by graded sucrose dehydration. OCT‐embedded tissues were cryosectioned at 12 μm thickness. After antigen retrieval for 2 h at 37°C, sections underwent PBS washes and blocking with 5% donkey serum (Beyotime, China) for 1 h. Sequential incubations were performed with validated primary antibodies: rabbit anti‐NOX4 (1:200; MA5‐32090, Invitrogen, USA), rabbit anti‐laminin (1:1000; ab11575, Abcam, UK) or rabbit anti‐CD68 polyclonal antibody (1:200, Signalway Antibody, USA) for 16 h at 4°C. After PBS rinsing, Donkey Anti‐Rabbit IgG H&L Alexa Fluor488 (1:800, ab150073, Abcam) was applied for 2 h. Nuclei were counterstained with DAPI before mounting with anti‐fade medium. Fluorescence imaging was executed on a Zeiss Axio Imager with standardized exposure settings. Muscle fibre CSA was analysed using ImageJ, with group comparisons based on biological replicates.

### 2.4 Immunochemical Staining

The GAS muscles fixed in 4% PFA were dehydrated through a graded alcohol series and embedded in OCT compound. Following cryosectioning (12 μm), the sections were dried for 2 h at 37°C. After washing with PBS to remove residual OCT, the sections were stained using a haematoxylin and eosin (H&E) staining kit (Beyotime). Images were captured using an optical microscope (Nikon Eclipse 80i) to assess inflammatory cell infiltration in the GAS.

### 2.5 ROS Determination

Fresh GAS muscles were embedded in OCT compound and sectioned into 10‐μm cryosections. Tissue sections were incubated with 5 μM dihydroethidium DHE (S0063, Beyotime) diluted in PBS for 30 min at room temperature under light‐protected conditions. After three 5‐min PBS washes, sections were mounted with antifade medium and imaged using a Zeiss Axio Imager M2 fluorescence microscope. DHE fluorescence intensity was analysed using ImageJ on representative tissue sections, with group comparisons based on biological replicates.

### 2.6 Ultrastructural Examination

GAS muscle samples were oriented perpendicular to myofibers and trimmed into cuboidal blocks (1 mm^2^ cross‐section). Tissues were fixed in 2.5% glutaraldehyde in phosphate buffer for 24 h at 4°C, followed by 1% osmium tetroxide (Sigma‐Aldrich, USA) post‐fixation for 2 h. After graded ethanol dehydration (30%–100%) and acetone clearance, samples were embedded in a low‐viscosity polymeric epoxy resin. Ultrathin sections (70 nm) on copper grids were stained with 1% uranyl acetate and lead citrate. Mitochondrial ultrastructures were observed under a transmission electron microscope (HT7700, Hitachi, Japan).

### 2.7 Real‐Time qPCR Analysis

Total RNA extraction was performed using TRIzol Reagent (15596026, Invitrogen), with cDNA synthesized from 1 μg RNA via HiScript III RT SuperMix (R323‐01, Vazyme, China) under 42°C for 15 min. qPCR amplification was conducted in triplicate using ChamQ Universal SYBR Master Mix (Q711‐03, Vazyme, China) on a BIO‐RAD system (BIO‐RAD‐96CFX) with the following programme: 95°C for 30 s; 40 cycles of 95°C for 10 s and 60°C for 30 s. Relative mRNA levels were calculated using the 2−ΔΔCt method. The primer sequences are listed in Table S1.

### 2.8 Mitochondrial Complex I/V Activity Assay

Mitochondrial complex I (NADH‐coenzyme Q reductase, CI) and complex V (F0F1‐ATPase/ATP synthase, CV) activities were determined using colorimetric assay kits (E‐bc‐K149‐M and E‐bc‐K153‐M; Elabscience, China) following manufacturer protocols. Absorbance measurements at 340 nm were conducted using a BioTek Synergy H1 microplate reader.

### 2.9 RIPK3 Overexpression in C2C12 Myoblasts

Murine C2C12 myoblasts (Chinese Academy of Sciences Cell Bank, China) were cultured in DMEM (Bio‐channel, China) supplemented with 10% FBS (Gibco, USA) and 1% penicillin–streptomycin (Beyotime). Cells were seeded in 24‐well plates at ~40% confluency (3 × 10^4^ cells/well) and transduced with a lentiviral vector (pcSLenti‐EF1‐EGFP‐P2A‐Puro‐CMV‐Ripk3‐3xFLAG‐WPRE; Obio Technology, China) at an optimized MOI in serum‐reduced medium for 16 h. After transduction, cells were cultured in complete medium for 72 h, followed by differentiation in DMEM containing 2% horse serum (Gibco, USA) with daily medium changes until myotube formation, after which protein was extracted.

### 2.10 Immunoblotting

Muscle tissues were homogenized using zirconium beads in a mechanical disruptor and lysed on ice for 30 min, whereas cultured cells were lysed in RIPA buffer. Both lysates were clarified by centrifugation (12 000 g, 15 min, 4°C), and protein concentrations were quantified using the BCA assay (Beyotime, China). Proteins were resolved on SDS‐PAGE gels and transferred to methanol‐activated PVDF membranes. After blocking with 5% BSA, membranes were incubated with primary antibodies against RIP3 (CST, #15828), MHC (R&D Systems, #MAB4470), TRIM63 (Signalway Antibody, #38580), Fbx32 (Abcam, #ab168372), FoxO3a (CST, #12829S), PGC‐1α (Abcam, #ab191838), Nrf2 (Abcam, #ab137550), p‐DRP1(Ser616) (CST, #4494), DRP1 (CST, #8570), MFF (CST, #84580), FIS1 (Proteintech, #10956‐1‐AP), NOX2 (Abcam, #ab129028), NOX4 (Invitrogen, #MA5‐32090) and GAPDH (BBI Life Sciences, #D110016), followed by incubation with HRP‐conjugated secondary antibodies (1:3000, Abcam, Cambridge, UK) for 2 h at room temperature. Signals were visualized using enhanced chemiluminescence reagent kits (Thermo Fisher, USA) and quantified using ImageJ software with GAPDH as the loading control.

### 2.11 Statistical Analysis

Statistical analyses were performed using GraphPad Prism (Version 9.0). Normality of data distribution was assessed using the Shapiro–Wilk test. For datasets that deviated from normality (p<0.05), logarithmic transformation was applied to achieve normality before analysis; all transformed datasets subsequently passed the normality test (p>0.05). Homogeneity of variances was evaluated using the Brown–Forsythe test, and all groups met the assumption of equal variances (p>0.05). For single‐factor experiments, one‐way ANOVA followed by Tukey's post hoc test was used to compare multiple groups. For two‐factor experiments, two‐way ANOVA was performed. When a significant interaction was detected (p<0.05), Tukey's post hoc test was used to compare all groups. When the interaction was not significant, but a significant main effect was present, planned comparisons between specific groups of interest (e.g., WT vs. KO within the same condition) were performed using Tukey's method to adjust for multiple testing. Results are expressed as mean ± standard deviation (SD). The statistical significance threshold was set at p<0.05. Detailed statistical parameters for all figures, including normality test results, ANOVA tables (sum of squares, degrees of freedom, mean squares, F‐values) and full post hoc comparison matrices, are provided in Table S2.

## 3 Results

### 3.1 Upregulation of RIPK3 in Denervated Muscle Atrophy

**FIGURE 1:**
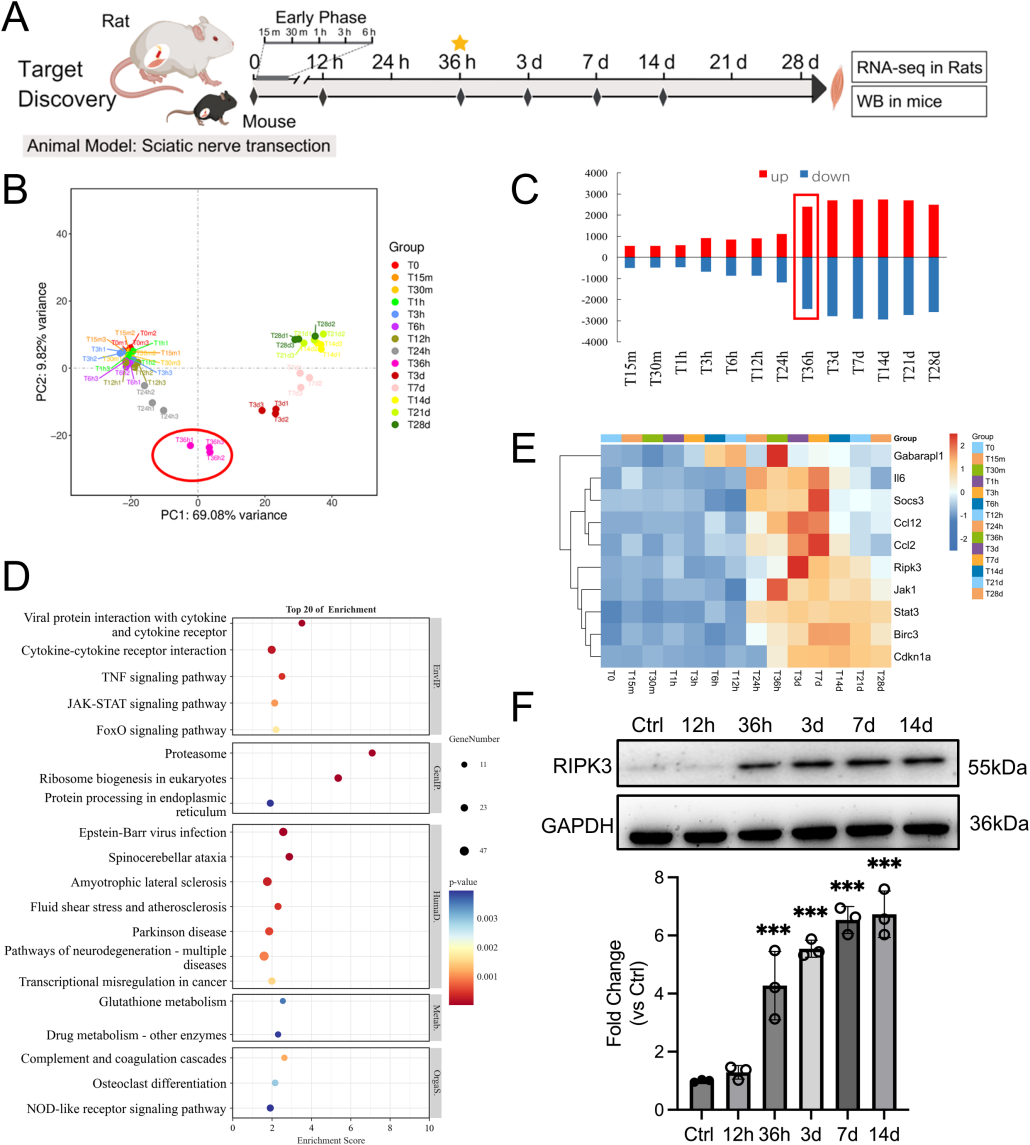
Temporal transcriptomic alterations in denervation‐induced muscle atrophy. (A) Schematic overview of the experimental strategy and timeline for target discovery. Transcriptomic screening was performed in denervated rats across multiple time points to identify early‐upregulated mediators, which were subsequently validated at the protein level in mice (created with MedPeer, medpeer.cn); (B) The principal component analysis (PCA) of transcriptional profiles, *n* = 3; (C) Quantifies differentially expressed genes (DEGs) across post‐denervation intervals; (D) KEGG pathway enrichment of upregulated genes at 36 h post‐denervation; (E) The expression patterns of 10 core regulatory genes through hierarchical clustering; (F) Time course of RIPK3 protein expression, with GAPDH as loading control, *n* = 3. Data are presented as mean ± SD and were analysed by one‐way ANOVA with Tukey's post hoc test; ***p < 0.001 versus Ctrl.

Transcriptomic profiling of denervated muscle atrophy demonstrated substantial gene expression changes in the target muscle at 36 h post‐denervation, characterized by a significant increase in differentially expressed genes (Figure 1A). These findings indicate that 36 h post‐denervation represents a pivotal time window for the initiation of denervated muscle atrophy (Figure 1B,C). KEGG pathway enrichment analysis revealed that the proteasome and cytokine–cytokine receptor interaction pathways were markedly activated at this time point, with concurrent coordinated upregulation of the TNF, FoxO, JAK–STAT and NOD‐like receptor signalling pathways (Figure 1D). Further network analysis identified 10 key regulatory genes—including Ripk3, Il6 and Stat3—that participate in multi‐pathway crosstalk. Our prior research has established that IL‐6 levels are markedly elevated following denervation, contributing to muscle atrophy via activation of the downstream JAK/STAT signalling cascade [31]. Notably, Ripk3 expression begins to shift at 36 h post‐denervation, showing a significant increase at 3 days and sustained elevation through 28 days (Figure 1E). Protein‐level analysis confirmed that RIPK3 abundance was significantly elevated as early as 36 h post‐denervation (Figure 1F), suggesting a potential role for RIPK3 as an early‐response mediator in the pathogenesis of denervated muscle atrophy, possibly through modulation of the inflammatory microenvironment.

### 3.2 RIPK3 Knockout Suppresses Proteolysis to Mitigate Denervation‐Induced Muscle Atrophy

**FIGURE 2:**
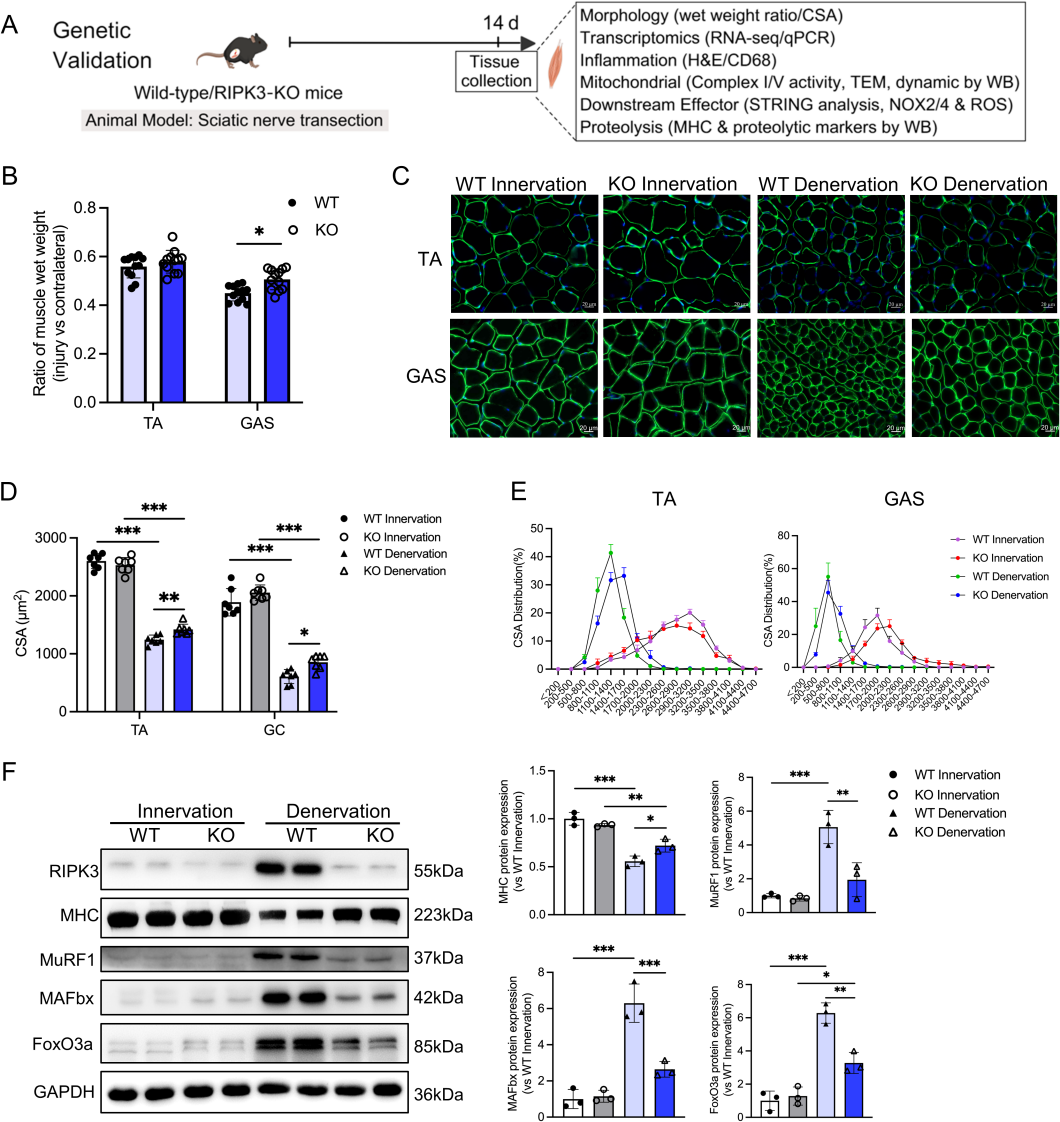
RIPK3 knockout inhibits excessive activation of the ubiquitin–proteasome system and ameliorates denervation‐induced muscle atrophy. (A) Schematic of the genetic validation strategy. RIPK3 knockout (RIPK3‐KO) mice and wild‐type (WT) littermates were subjected to sciatic nerve denervation for 14 days. Gastrocnemius and tibialis anterior muscles were collected for morphological, transcriptomic and molecular analyses to assess the functional role of RIPK3 (created with MedPeer, medpeer.cn); (B) Wet weight ratio of tibialis anterior and gastrocnemius muscles, *n* = 12; (C) Laminin staining of cross‐sectional area of tibialis anterior and gastrocnemius muscles, scale bar = 20 μm; (D) Statistical analysis of cross‐sectional area of muscle fibres in tibialis anterior and gastrocnemius muscles, *n* = 7, for tibialis anterior CSA, data were log‐transformed to meet normality and homogeneity of variances assumptions; statistical analyses were performed on transformed data, whereas figures display untransformed data for clarity; (E) Distribution of muscle fibres in tibialis anterior and gastrocnemius muscles, *n* = 5; (F) Western blot analysis of protein levels of RIPK3, MHC, MuRF1, MAFbx and FoxO3a in gastrocnemius muscle, with GAPDH as loading control, *n* = 3. FoxO3a migrates as a doublet, reflecting known protein variants or modifications. All data are presented as mean ± SD and were analysed by two‐way ANOVA with Tukey's post hoc test; *p < 0.05, **p < 0.01, ***p < 0.001.

To investigate RIPK3's role in muscle atrophy, WT and RIPK3‐KO mice were assessed 14 days post‐denervation (Figure 2A). Wet weight ratio analysis using two‐way ANOVA (muscle × genotype) revealed no significant interaction (*p* = 0.1448), but significant main effects of muscle (*p* < 0.001) and genotype (*p* = 0.0031; Figure 2B). Post hoc analysis showed that RIPK3 knockout significantly increased wet weight ratio in GAS (*p* = 0.0110), but not in TA (*p* = 0.6520). Laminin staining demonstrated larger myofiber CSA in both muscles of KO mice (Figure 2C–E). For GAS CSA, two‐way ANOVA showed significant surgery and genotype effects (both *p* < 0.01), with KO mice exhibiting larger CSA after denervation (Den‐WT vs. Den‐KO: *p* = 0.04). For TA CSA, two‐way ANOVA on log‐transformed data revealed a significant interaction (*p* = 0.0021) and genotype effect (*p* < 0.001), with KO attenuating CSA loss after denervation (Den‐WT vs. Den‐KO: *p* = 0.0030). Western blotting revealed significant interactions for the structural protein MHC, the transcription factor FoxO3a and the E3 ubiquitin ligases MuRF1 and MAFbx (all *p* < 0.01; Figure 2F). Post hoc analysis confirmed that denervation‐induced changes were significantly attenuated in KO mice compared with WT (Den‐WT vs. Den‐KO: MHC *p* = 0.0278, FoxO3a *p* = 0.0012, MuRF1 *p* = 0.0030, MAFbx *p* < 0.001). These results demonstrate that RIPK3 knockout inhibits FOXO3‐driven ubiquitin–proteasome proteolysis, mitigating denervation‐induced muscle atrophy.

### 3.3 RIPK3 Knockout Mitigates Muscle Atrophy via Suppression of Inflammation

**FIGURE 3:**
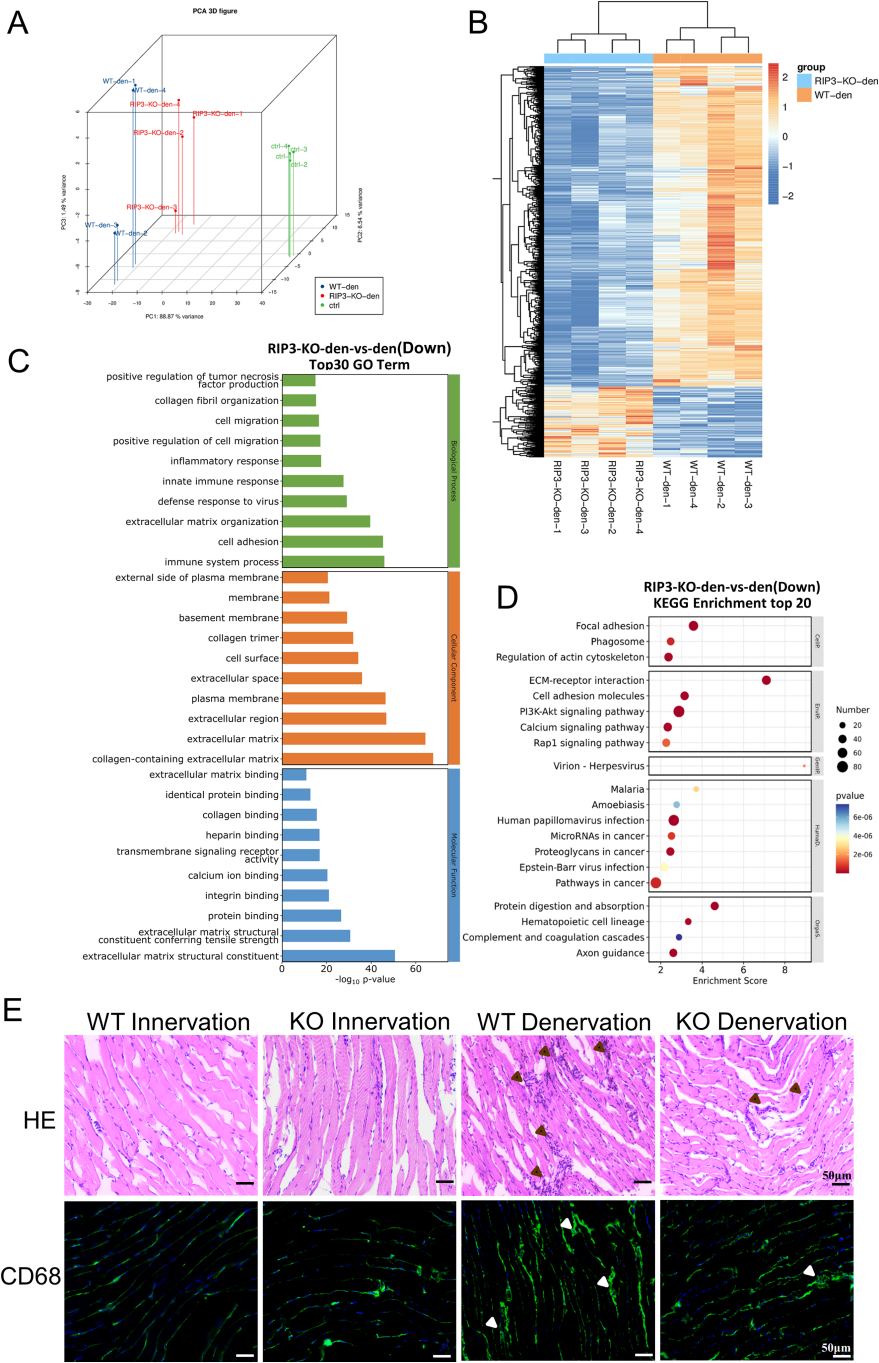
RIPK3 knockout mitigates inflammation in denervated muscle atrophy. (A) PCA of global gene expression profiles, *n* = 4; (B) Heatmap visualization of differentially expressed genes (|log2FC| > 1, *q* < 0.05); (C) GO enrichment of downregulated genes; (D) KEGG pathway analysis of downregulated transcriptional signatures; (E) Histological evaluation by H&E staining and CD68^+^ macrophage infiltration analysis via immunofluorescence, scale bar = 50 μm.

To investigate RIPK3‐mediated molecular mechanisms in muscle wasting, we performed RNA‐seq on denervated GAS muscles from KO and WT mice. PCA revealed complete separation of transcriptional profiles between RIPK3‐KO and WT cohorts following denervation (Figure 3A). Comparative analysis identified 1554 significantly downregulated and 343 upregulated genes in KO mice versus WT controls (Figure 3B). GO enrichment analysis demonstrated predominant downregulation of biological processes including immune response, inflammatory activation and extracellular matrix (ECM)‐related cellular components/molecular functions (Figure 3C). KEGG pathway analysis highlighted suppression of ECM‐receptor interactions and cell adhesion molecules (Figure 3D). Histopathological analysis revealed reduced inflammatory cell infiltration in RIPK3‐KO muscles, consistent with attenuated macrophage recruitment as evidenced by CD68^+^ immunostaining (Figure 3E). These findings collectively indicate that RIPK3 knockout mitigates denervation‐induced inflammatory cascades in skeletal muscle.

### 3.4 RIPK3 Knockout Mitigates Muscle Atrophy via Activation of OXPHOS

**FIGURE 4:**
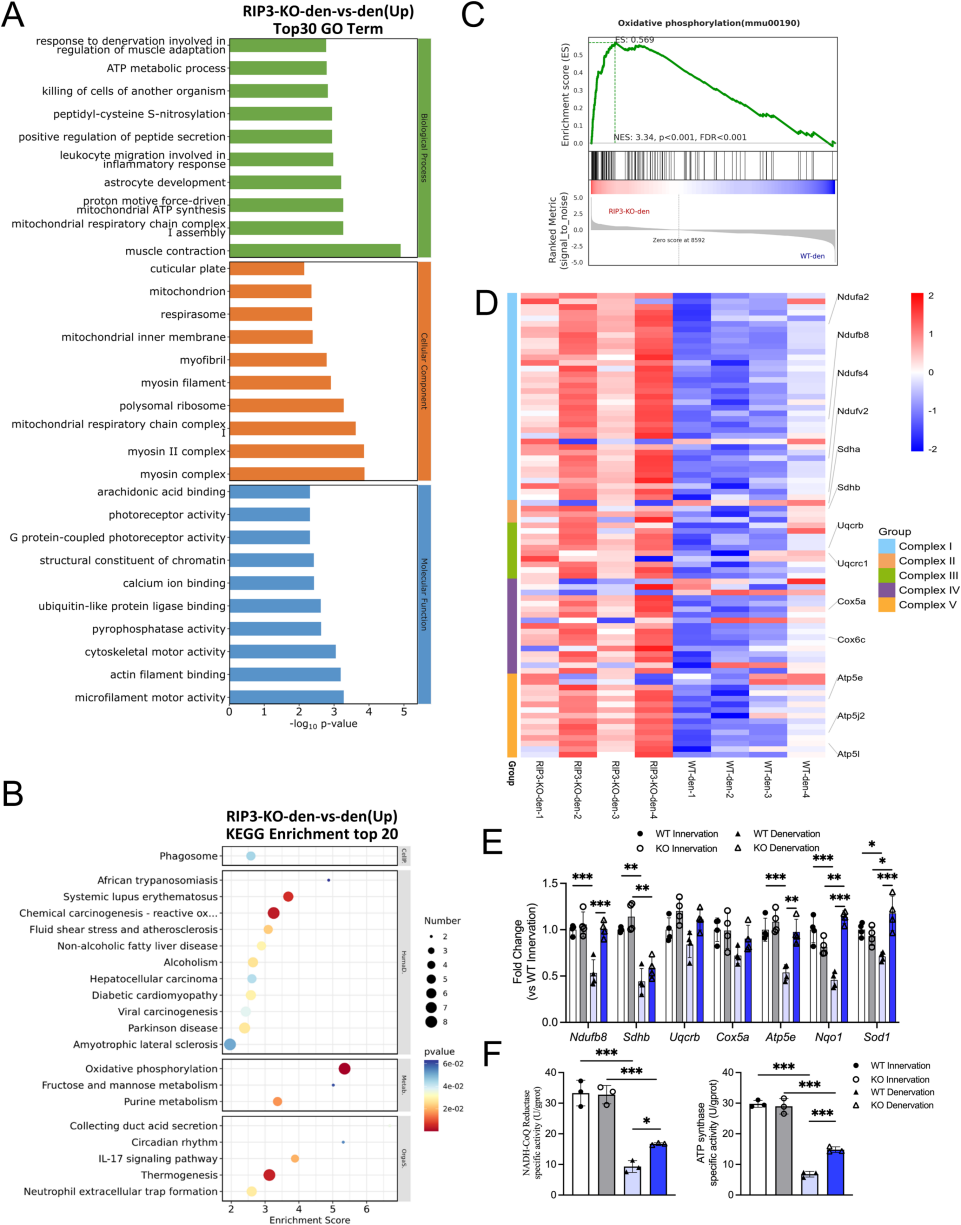
RIPK3 knockout modulates mitochondrial oxidative phosphorylation in denervated muscle atrophy. (A) GO enrichment of upregulated genes; (B) KEGG pathway enrichment analysis of upregulated genes; (C) GSEA confirming oxidative phosphorylation (OXPHOS) pathway activation; (D) Heatmap visualization of nuclear‐encoded OXPHOS‐related gene expression. Genes are organized according to their respective mitochondrial respiratory chain complexes (I–V). To focus the analysis on clear expression trends, mitochondrial DNA‐encoded genes, non‐canonical components and genes with minimal expression were excluded; (E) qRT‐PCR analysis of mitochondrial electron transport chain components (*Ndufb8*, *Sdhb*, *Uqcrb*, *Cox5a*, *Atp5e*) and oxidative stress‐response markers (*Nqo1*, *Sod1*), *n* = 4; (F) Enzyme activity assays for respiratory chain complexes I (NADH dehydrogenase) and V (ATP synthase), *n* = 3. All data are presented as mean ± SD and were analysed by two‐way ANOVA with Tukey's post hoc test; *p < 0.05, **p < 0.01, ***p < 0.001.

GO analysis of upregulated genes revealed enhanced enrichment in muscle contraction, mitochondrial functions (including respiratory complex I assembly and ATP metabolic process) and cytoskeletal organization (Figure 4A). KEGG pathway analysis demonstrated significant enrichment of oxidative phosphorylation pathways alongside the ‘Chemical carcinogenesis‐reactive oxygen species’ pathway, featuring shared induction of mitochondrial complex‐associated and antioxidant genes (Figure 4B). Consistent with transcriptomic findings, GSEA confirmed upregulation of oxidative phosphorylation genes in RIPK3‐KO mice (Figure 4C,D). This pathway coordinates electron transport through complexes I–IV to power ATP synthase (complex V), constituting the core bioenergetic mechanism for maintaining mitochondrial homeostasis [32]. Given that complex I (as the major entry point of the electron transport chain) and complex V (as the terminal ATP producer) are established key nodes frequently impaired in muscle wasting conditions [33], and considering their specific enrichment in our GO analysis, we prioritized these two complexes for functional validation. qPCR analysis showed that RIPK3 knockout significantly upregulated genes involved in mitochondrial function (*Ndufb8*, *Atp5e*) and antioxidant defence (*Nqo1*, *Sod1*) after denervation. Two‐way ANOVA revealed significant interactions for all four genes (all *p* < 0.05), and post hoc analysis confirmed that their expression was significantly higher in KO mice than in WT mice under denervated conditions (Den‐WT vs. Den‐KO: *Ndufb8 p* < 0.001, *Atp5e p* = 0.0011, *Nqo1 p* < 0.001, *Sod1 p* < 0.001; Figure 4E). Main effects of surgery and/or genotype were also observed for these genes (see Table S2 for detailed statistics). Other tested subunits showed concordant but non‐significant trends. Functional assays demonstrated that RIPK3 knockout specifically rescued denervation‐induced impairments in mitochondrial complex I and V activities (Figure 4F). Two‐way ANOVA revealed significant interactions for both complex I (*p* = 0.0373) and complex V (*p* = 0.0011), with a significant main effect of genotype for complex V (*p* = 0.0034). Post hoc analysis confirmed that after denervation, KO mice exhibited significantly higher complex I (*p* = 0.0438) and complex V (*p* < 0.001) activities compared with WT mice. These findings indicated that RIPK3 deficiency enhances mitochondrial metabolism, primarily through restoring the function of key respiratory complexes I and V, to counteract denervation‐induced muscle atrophy.

### 3.5 RIPK3 Knockout Mitigates Muscle Atrophy via Restoration of Mitochondrial Homeostasis

**FIGURE 5:**
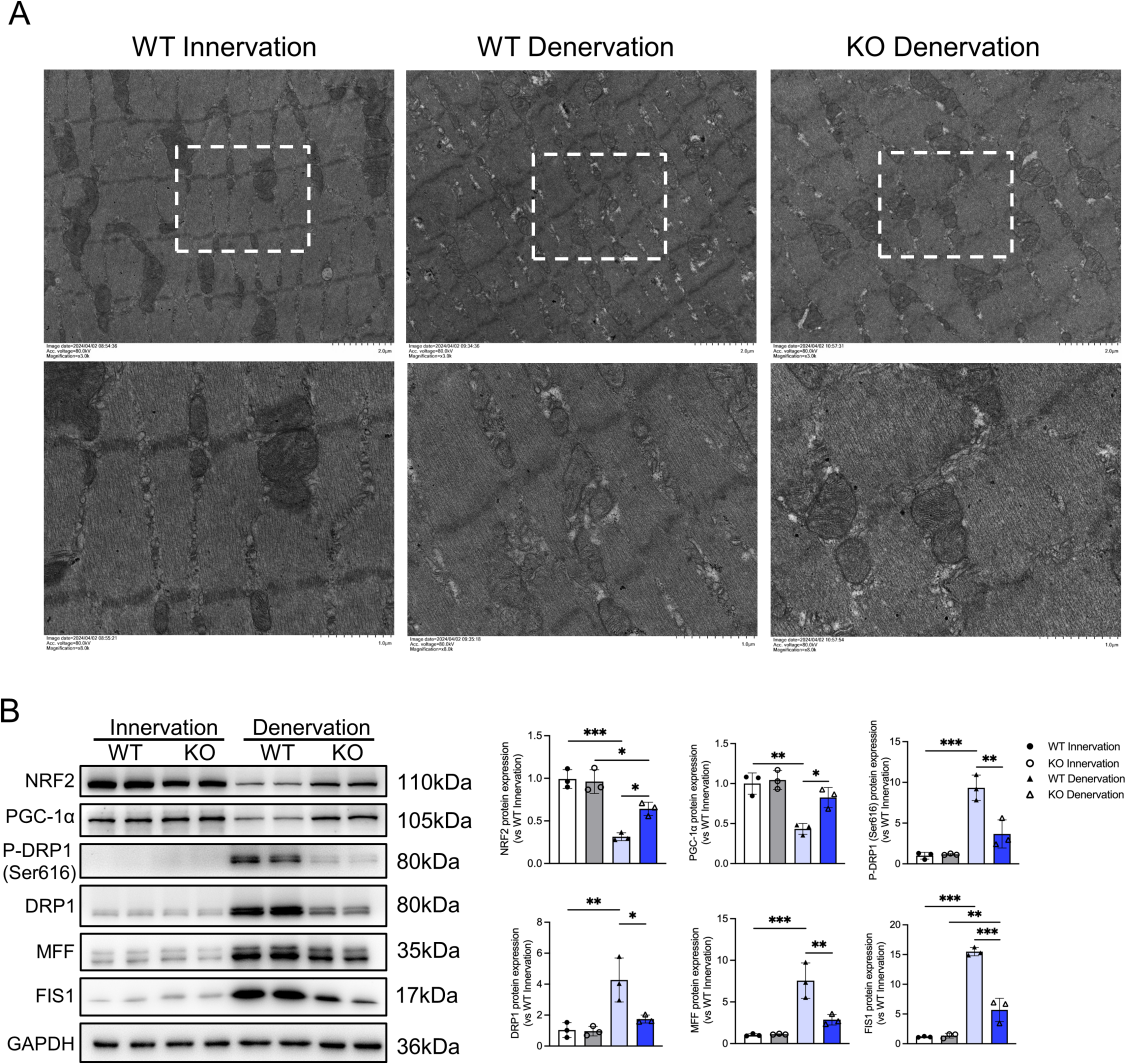
RIPK3 knockout ameliorates mitochondrial ultrastructural abnormalities in denervated muscle atrophy. (A) Transmission electron microscopy revealing mitochondrial ultrastructure in gastrocnemius. (B) Western blot quantification of biogenesis marker PGC‐1α and NRF2, fission regulators FIS1, MFF, DRP1 and phospho‐DRP1 (p‐DRP1), with GAPDH loading control, *n* = 3. DRP1 and p‐DRP1 migrate as a doublet, reflecting known protein variants or modifications. All data are presented as mean ± SD and were analysed by two‐way ANOVA with Tukey's post hoc test; *p < 0.05, **p < 0.01, ***p < 0.001.

Building on the observed OXPHOS activation, we investigated whether RIPK3 knockout improves mitochondrial integrity to mitigate muscle atrophy. Transmission electron microscopy revealed severe cristae disruption and mitochondrial fragmentation in denervated wild‐type muscle, whereas RIPK3‐KO mice exhibited preserved mitochondrial ultrastructure (Figure 5A). Given that mitochondrial homeostasis depends on balanced dynamics (fission/fusion) and quality control mechanisms [34], we analysed key regulatory proteins (Figure 5B). Two‐way ANOVA revealed significant interactions for all six proteins assessed (NRF2: *p* = 0.0143; PGC‐1α: *p* = 0.0302; p‐DRP1: *p* = 0.0027; DRP1: *p* = 0.0256; FIS1: *p* < 0.001; MFF: *p* = 0.0058). Post hoc analysis confirmed that RIPK3 knockout significantly elevated the biogenesis markers NRF2 (Den‐WT vs. Den‐KO: *p* = 0.0159) and PGC‐1α (Den‐WT vs. Den‐KO: *p* = 0.0129) while suppressing fission‐related components after denervation, including p‐DRP1 (*p* = 0.0017), DRP1 (*p* = 0.0171), FIS1 (*p* < 0.001) and MFF (*p* = 0.0037). These data collectively demonstrate that RIPK3 deficiency is associated with restored mitochondrial functional integrity through enhanced biogenesis and suppressed fission, providing mechanistic insights into its protective effects against denervation‐induced atrophy.

### 3.6 RIPK3 Knockout Mitigates Muscle Atrophy via Suppression of NOX4‐ROS Axis

**FIGURE 6:**
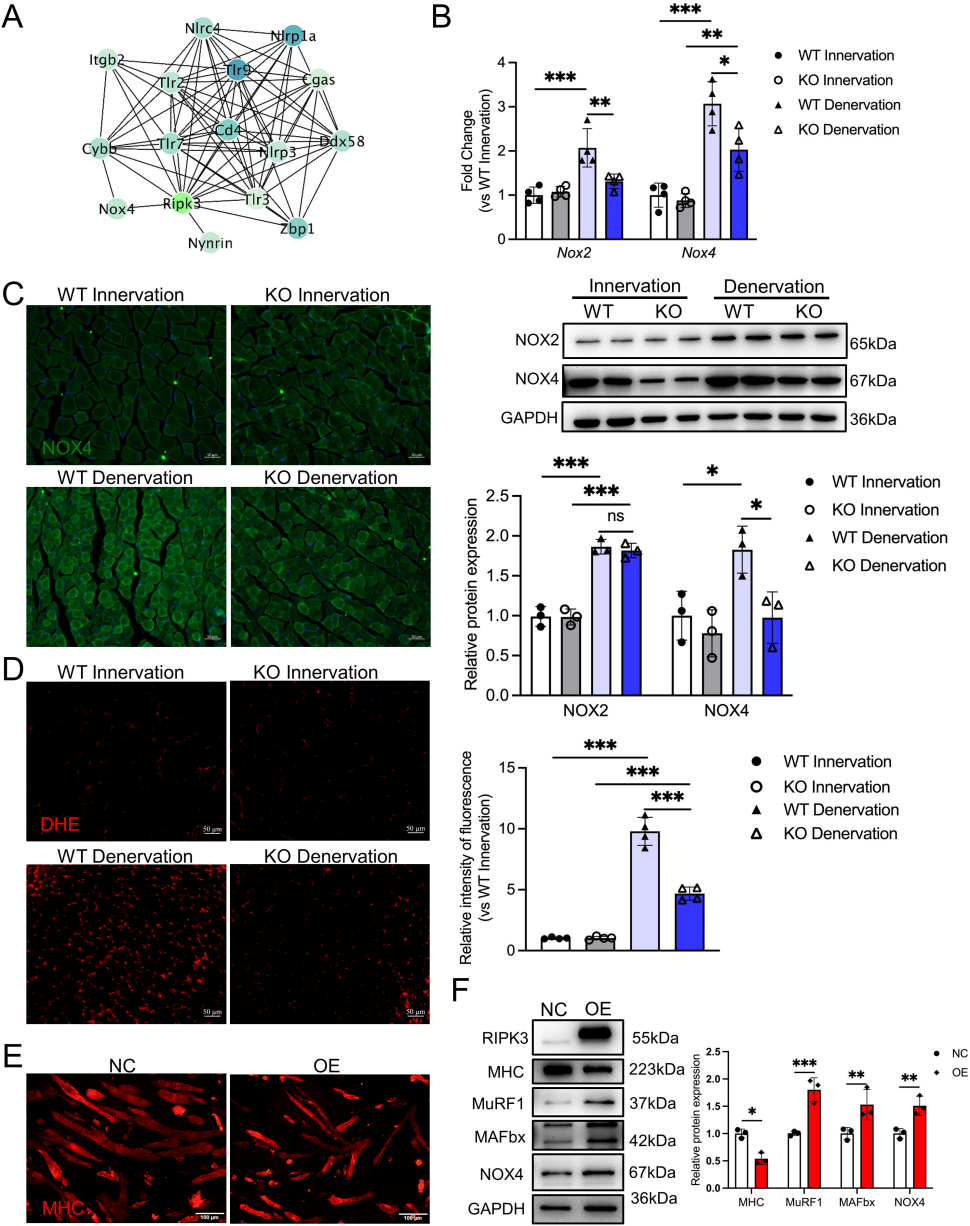
Downstream targets mediating RIPK3‐regulated mitochondrial homeostasis. (A) STRING protein interaction network; (B) Validation of Nox2 and Nox4 expression by qRT‐PCR (mRNA, *n* = 4) and Western blot (protein, *n* = 3); (C) Immunofluorescence staining of NOX4 in gastrocnemius muscle sections, scale bar = 50 μm; (D) Representative images and quantification of DHE staining for ROS in gastrocnemius muscle sections; scale bar = 50 μm, *n* = 4, statistical analysis was performed on log‐transformed data to meet ANOVA assumptions; figures display untransformed data for clarity; (E) Immunofluorescence staining of MHC of C2C12 myotubes, scale bar = 100 μm. (F) Western blot quantification of MHC, MuRF1, MAFbx and NOX4, with GAPDH as loading control, *n* = 3. The specific MAFbx band is indicated at ~42 kDa; the upper band is non‐specific. All data are presented as mean ± SD and were analysed by two‐way ANOVA with Tukey's post hoc test; *p < 0.05, **p < 0.01, ***p < 0.001, NS: non‐significant.

To mechanistically link RIPK3 deficiency with mitochondrial protection, we mapped its downstream effectors through STRING‐based interactome analysis, identifying redox regulators NOX4 and NOX2 (Cybb) as direct network nodes (Figure 6A). qPCR analysis revealed that RIPK3 knockout significantly attenuated denervation‐induced NOX4 mRNA upregulation (interaction: *p* = 0.0336; Den‐WT vs. Den‐KO: *p* = 0.011), whereas NOX2 mRNA showed a significant interaction (*p* = 0.0070) but no genotype effect in post hoc comparisons (Den‐WT vs. Den‐KO: *p* = 0.0062 for NOX2; Figure 6B). At the protein level, RIPK3 knockout selectively reduced NOX4 expression after denervation (interaction: *p* = 0.1105; Den‐WT vs. Den‐KO: *p* = 0.0366), with no effect on NOX2 (interaction: *p* = 0.7408; Den‐WT vs. Den‐KO: *p* = 0.9378; Figure 6C,D). Consistent with NOX4 suppression, DHE staining showed that RIPK3 knockout markedly reduced denervation‐induced ROS accumulation (two‐way ANOVA on log‐transformed data: interaction *p* < 0.001; Den‐WT vs. Den‐KO: *p* < 0.001; Figure 6E). Complementary overexpression studies in C2C12 myotubes confirmed that RIPK3 directly enhances NOX4 expression (NC vs. OE: *p* = 0.0045), along with the atrophy markers MuRF1 (*p* < 0.001) and MAFbx (*p* = 0.0097), while reducing MHC levels (*p* = 0.0307; Figure 6F,G). These findings establish a regulatory link between RIPK3 and the NOX4‐ROS signalling axis in denervation‐induced muscle atrophy.

### 3.7 Pharmacological RIPK3 Inhibition Mitigates Muscle Atrophy

**FIGURE 7:**
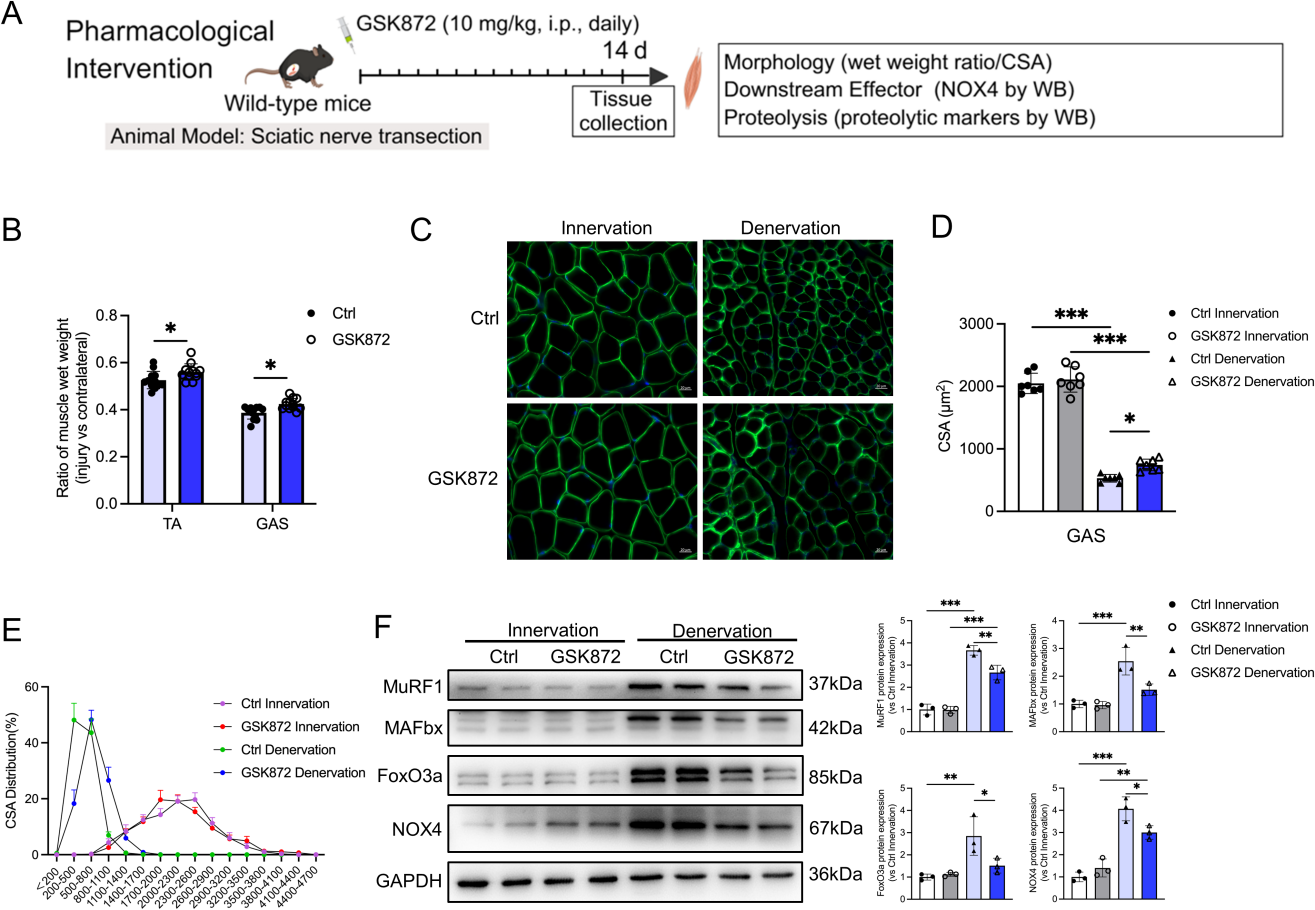
Pharmacological RIPK3 inhibition attenuates denervation‐induced muscle atrophy. (A) Schematic of the pharmacological validation strategy. Following sciatic nerve denervation, mice received daily intraperitoneal injections of the selective RIPK3 inhibitor GSK872 (10 mg/kg) or vehicle control for 14 days. Muscles were harvested for morphological and molecular analyses to evaluate therapeutic efficacy (created with MedPeer, medpeer.cn); (B) Wet weight ratio of tibialis anterior and gastrocnemius muscles, *n* = 12; (C) Laminin staining of cross‐sectional area of gastrocnemius muscles, scale bar = 20 μm; (D) Statistical analysis of cross‐sectional area of muscle fibres in gastrocnemius muscles, *n* = 7; (E) Distribution of muscle fibres in gastrocnemius muscles, *n* = 5; (F) Western blot analysis of protein levels of MuRF1, MAFbx, FoxO3a and NOX4 in gastrocnemius muscle, with GAPDH as the internal reference, *n* = 3. FoxO3a migrates as a doublet, reflecting known protein variants or modifications. All data are presented as mean ± SD and were analysed by two‐way ANOVA with Tukey's post hoc test; *p < 0.05, **p < 0.01, ***p < 0.001.

To validate the therapeutic potential of RIPK3 modulation, we conducted pharmacological intervention using intraperitoneal administration of a selective RIPK3 inhibitor (Figure 7A). Wet weight ratio analysis (two‐way ANOVA: interaction *p* = 0.8803, treatment effect *p* = 0.0003) showed that GSK872 significantly attenuated mass loss in both TA (Ctrl vs. GSK872: *p* = 0.0467) and GAS (Ctrl vs. GSK872: *p* = 0.0277; Figure 7B). CSA analysis of GAS muscle revealed a significant treatment effect (*p* = 0.0179) with preserved myofiber size after denervation (Den‐Ctrl vs. Den‐GSK872: *p* = 0.0478; Figure 7C–E). Mechanistically, GSK872 treatment significantly attenuated denervation‐induced upregulation of FOXO3a (interaction: *p* = 0.0281; Den‐Ctrl vs. Den‐GSK872: *p* = 0.0347), MuRF1 (interaction: *p* = 0.0086; Den‐Ctrl vs. Den‐GSK872: *p* = 0.0047), MAFbx (interaction: *p* = 0.0170; Den‐Ctrl vs. Den‐GSK872: *p* = 0.0095) and NOX4 (interaction: *p* = 0.0111; Den‐Ctrl vs. Den‐GSK872: *p* = 0.0398; Figure 7F). The treatment main effect was not significant for FOXO3a (*p* = 0.0568) and NOX4 (*p* = 0.1788), consistent with the specific inhibitory action of GSK872 under pathological conditions without affecting baseline levels. These pharmacological findings corroborate the genetic evidence, supporting RIPK3 targeting as a viable strategy against denervation induced muscle atrophy.

## 4 Discussion

Skeletal muscle functions as a crucial rheostat for proteostatic control and systemic metabolism, with its atrophy developing through multi‐layered pathomechanistic networks. Current pharmacotherapeutic strategies continue to face significant barriers, particularly disease subtype variability and therapeutic resistance arising from signalling redundancy [13]. Neurogenic muscular atrophy presents particular clinical urgency, given its aggressive pathophysiology and unsatisfactory rehabilitation outcomes post‐microsurgery. This knowledge gap underscores the necessity for mechanistic deconvolution of critical regulatory hubs. Our investigation synergized genome‐wide expression analytics with RIPK3 knockout neurectomy models, mechanistically decoding this kinase's dual regulation of mitochondrial quality control and neuroinflammatory pathways in denervation‐induced muscle wasting.

Our temporal transcriptomic mapping delineates the 36‐h post‐injury phase as a critical transition point for neurogenic muscle wasting, characterized by maximal differential gene expression. This analytical approach enabled identification of *Il6*, *Stat3*, *Socs3* and *Ripk3* as principal inflammatory modulators through KEGG pathway enrichment of temporally upregulated targets. Building on our earlier discovery of IL‐6‐mediated JAK/STAT3 pathway hyperactivation in muscular wasting, intramuscular administration of exogenous IL‐6 was shown to exacerbate atrophy by dual mechanisms: activating immune receptors while suppressing energy metabolism [31, 35]. Complementing these findings, *Socs3* expression dynamics demonstrate an inverse correlation with both STAT3 phosphorylation intensity and histological inflammation metrics, reinforcing its role as a compensatory brake in cytokine‐driven atrophy [36]. Although *Cdkn1a*/p21 merits investigation for its HDAC4‐mediated atrophic potential [30], our functional prioritization of RIPK3 stems from its unique temporal expression profile exhibiting sustained upregulation from initial denervation through progressive atrophy stages. This persistent activation pattern suggests RIPK3 operates as a molecular amplifier rather than a transient responder in neurogenic muscle deterioration. Functional validation demonstrated RIPK3 ablation markedly ameliorated atrophy phenotypes and attenuated macrophage infiltration.

The inflammatory microenvironment exacerbates atrophy through dual mechanisms: suppressing mTOR‐mediated myofibrillar synthesis while activating proteolytic degradation via the ubiquitin–proteasome system. Clinical evidence correlates elevated circulating inflammatory cytokines with muscular wasting across sepsis, neoplasia and diabetes, underscoring inflammatory cascades as central pathological drivers [37]. Mechanistically, cytokines like TNF‐α induce MAFbx/MuRF1 expression via NF‐κB/p38‐MAPK axis activation, whereas FOXO3 nuclear translocation synchronizes transcription of multiple atrogenes [32]. Our data revealed RIPK3 deficiency significantly downregulated FoxO3a and its targets MAFbx/MuRF1, suggesting RIPK3 exacerbates denervation atrophy through inflammatory‐proteolytic coupling.

RIPK3 signalling, canonically associated with MLKL‐dependent necroptosis, exhibits multifaceted regulatory roles spanning inflammasome activation, metabolic reprogramming and mitochondrial homeostasis. Transcriptomic profiling of denervated GAS revealed RIPK3 ablation concurrently attenuated inflammatory pathways and enhanced oxidative phosphorylation, evidenced by restored mitochondrial complex I/V activity and cristae integrity via TEM ultrastructural analysis. These findings position RIPK3 as a rheostat coordinating energy metabolism and inflammatory responses in atrophic muscle. Mechanistically, RIPK3 deficiency reversed denervation‐induced suppression of the PGC‐1α/NRF2 axis while inhibiting DRP1(S616)‐mediated fission through reduced FIS1/MFF recruitment, a dual modulation strategy counteracting mitochondrial fragmentation and bioenergetic collapse [38]. Given mitochondrial dysfunction's established role as both cause and consequence of inflammatory atrophy, our work delineates a self‐reinforcing RIPK3‐driven circuit linking neurogenic inflammation to organelle destabilization. The therapeutic implications of targeting this pathway extend beyond muscular preservation, potentially offering strategies to uncouple metabolic insufficiency from chronic inflammatory states.

To delineate RIPK3's downstream effectors, we performed STRING network analysis revealing robust RIPK3‐NOX4/NOX2 co‐expression patterns. Western validation demonstrated RIPK3 ablation selectively reduced NOX4 protein levels without affecting NOX2. The RIPK3‐NOX4 regulatory specificity was further validated through two experimental paradigms—RIPK3 overexpression in C2C12 myotubes elevated NOX4 expression, whereas in vivo pharmacological RIPK3 inhibition exerted the opposite regulatory effect. Critically, RIPK3 knockout muscles exhibited diminished ROS generation paralleling NOX family downregulation. Given NOX4’s mitochondrial localization and enzymatic ROS production, we propose its central role in denervation‐induced oxidative cascades. Neural interruption triggers ischemic mitochondrial uncoupling, heightening NOX activity. RIPK3‐mediated NOX4 amplification drives pathological ROS surges that disrupt bioenergetic flux and activate proteolytic systems. Substantiating this model, RIPK3 deficiency attenuated both DRP1 expression and phosphorylation at Ser616—a known NOX4 target site coordinating mitochondrial hyperfission [39]. Co‐immunoprecipitation assays in MLE‐12 cells have confirmed endogenous interaction between NOX4 and DRP1, forming a functional protein complex [40]. Given the observed mitochondrial fragmentation phenotype in our atrophy models, coupled with emerging evidence linking NOX4‐derived ROS to DRP1 activation, future studies should mechanistically interrogate the RIPK3‐NOX4‐DRP1 signalling axis as a potential amplifier of denervation‐induced bioenergetic crisis. Our mechanistic focus on the GAS was based on its robust phenotypic response, which provided a clear system for pathway discovery. RIPK3 knockout significantly increased CSA in both GAS and TA after denervation, confirming the protective effect in both muscle types. Although the wet weight ratio showed significant preservation in GAS, the increase in TA did not reach statistical significance in the genetic model. However, pharmacological RIPK3 inhibition significantly improved the wet weight ratio in both muscles, further supporting the general relevance of this pathway. The subtle differences between muscle types and experimental conditions may reflect biological variability or sample size considerations. We recognize that the sample size for some molecular analyses is relatively modest, and future studies with larger cohorts will help further validate these findings.

Collectively, our findings demonstrate that denervation‐induced RIPK3 activation drives skeletal muscle atrophy through a NOX4‐mediated axis that disrupts mitochondrial bioenergetics, amplifies inflammation and activates proteolytic systems. The therapeutic efficacy of both genetic and pharmacological RIPK3 inhibition supports its potential as a strategic intervention target for neurogenic atrophy. Notably, the core pathology we delineate—RIPK3‐driven mitochondrial dysfunction, oxidative stress and inflammatory‐proteolytic coupling—operates at the intersection of pathways well documented in diverse muscle wasting conditions, including cancer cachexia and sarcopenia [6, 7, 8, 9]. RIPK3 has been directly implicated in relevant preclinical models: it mediates myofibre death in dystrophin‐deficient mice and contributes to myopathy in Duchenne muscular dystrophy [28, 29], both of which share features with cachexia and sarcopenia. The RIPK3‐NOX4 axis identified here may therefore represent a conserved mechanism across different wasting etiologies, although direct testing in cachexia and sarcopenia models will be essential to confirm this possibility. Future investigations in tumour‐bearing or aged mice will determine whether RIPK3 inhibition confers protection through suppression of NOX4 and restoration of mitochondrial homeostasis. Collectively, our study establishes a mechanistic foundation for targeting RIPK3 in neurogenic atrophy and provides a rationale for exploring its broader relevance to other muscle wasting disorders.

## Funding

This work was supported by the National Natural Science Foundation of China (Nos. 32130060, 82401633, 82072160), the Nantong Science and Technology Bureau (No. JC2024085), the Natural Science Foundation of Jiangsu Province (No. BK20232023), the Natural Science Research Projects in Universities of Jiangsu Province (Nos. 24KJA310007, 24KJB310013) and the Suzhou Science and Technology Development Program (No. SYW2024048).

## Ethics Statement

All experimental protocols were conducted in strict accordance with the Animal Care Guidelines of Nantong University and were approved by the Jiangsu Provincial Laboratory Animal Management Committee (Approval No. S20230409‐006 for rats and No. S20230709‐006 for mice).

## Conflicts of Interest

The authors declare no conflicts of interest.

## Supporting information


**Data S1:** Supporting information.

**Table S1:** Primer sequence for genes.
**Table S2:** Statistical summary.

## References

[1] L. Yin , N. Li , W. Jia , et al., “Skeletal Muscle Atrophy: From Mechanisms to Treatments,” Pharmacological Research 172 (2021): 105807. 34389456 10.1016/j.phrs.2021.105807

[2] Y. Ji , Q. Jiang , B. Chen , et al., “Endoplasmic Reticulum Stress and Unfolded Protein Response: Roles in Skeletal Muscle Atrophy,” Biochemical Pharmacology 234 (2025): 116799. 39952329 10.1016/j.bcp.2025.116799

[3] J. Yi , J. Chen , X. Yao , et al., “Myokine‐Mediated Muscle‐Organ Interactions: Molecular Mechanisms and Clinical Significance,” Biochemical Pharmacology 242 (2025): 117326. 40957493 10.1016/j.bcp.2025.117326

[4] J. Sun , H. Zhou , Z. Chen , et al., “Altered m6A RNA Methylation Governs Denervation‐Induced Muscle Atrophy by Regulating Ubiquitin Proteasome Pathway,” Journal of Translational Medicine 21 (2023): 845. 37996930 10.1186/s12967-023-04694-3 PMC10668433

[5] L. Qi , F. Zhang , K. Wang , et al., “Advancements in Skeletal Muscle Tissue Engineering: Strategies for Repair and Regeneration of Skeletal Muscle Beyond Self‐Repair,” Regenerative Biomaterials 12 (2025): rbaf050. 40599408 10.1093/rb/rbaf050 PMC12212644

[6] Q. Li , X. Yin , W. Wan , et al., “EIF4A3 Promotes Muscle Atrophy and Aging by Inhibiting the FAK Pathway Through NEDD9 mRNA Destabilization,” Journal of Cachexia, Sarcopenia and Muscle 16 (2025): e70010. 40641186 10.1002/jcsm.70010 PMC12246388

[7] X. A. Zhu , S. Starosta , M. Ferrer , et al., “A Neuroimmune Circuit Mediates Cancer Cachexia‐Associated Apathy,” Science 388 (2025): eadm8857. 40208971 10.1126/science.adm8857 PMC13051291

[8] X. Chen , Y. Ji , R. Liu , et al., “Mitochondrial Dysfunction: Roles in Skeletal Muscle Atrophy,” Journal of Translational Medicine 21 (2023): 503. 37495991 10.1186/s12967-023-04369-z PMC10373380

[9] Y. Ji , M. Li , M. Chang , et al., “Inflammation: Roles in Skeletal Muscle Atrophy,” Antioxidants (Basel) 11, no. 9 (2022): 1686. 36139760 10.3390/antiox11091686 PMC9495679

[10] F. Ribeiro , P. R. Jannig , S. Labeit , and A. S. Moriscot , “Small‐Molecule Targeting MuRF1 Protects Against Denervation‐Induced Diaphragmatic Dysfunction: Underlying Molecular Mechanisms,” Journal of Cachexia, Sarcopenia and Muscle 16 (2025): e70119. 41243395 10.1002/jcsm.70119 PMC12620420

[11] J. Wu , Q. Han , D. Gui , and Y. Qian , “Multidimensional Advances in Neural Interface Technology for Peripheral Nerve Repair: From Material Innovation to Clinical Translation,” Materials Today Bio 34 (2025): 102092. 10.1016/j.mtbio.2025.102092 PMC12296523 40727303

[12] X. Yao , T. Xue , B. Chen , et al., “Advances in Biomaterial‐Based Tissue Engineering for Peripheral Nerve Injury Repair,” Bioactive Materials 46 (2025): 150–172. 39760068 10.1016/j.bioactmat.2024.12.005 PMC11699443

[13] G. Sirago , M. A. Pellegrino , R. Bottinelli , M. V. Franchi , and M. V. Narici , “Loss of Neuromuscular Junction Integrity and Muscle Atrophy in Skeletal Muscle Disuse,” Ageing Research Reviews 83 (2023): 101810. 36471545 10.1016/j.arr.2022.101810

[14] Y. Shen , R. Zhang , L. Xu , et al., “Microarray Analysis of Gene Expression Provides New Insights Into Denervation‐Induced Skeletal Muscle Atrophy,” Frontiers in Physiology 10 (2019): 1298. 31681010 10.3389/fphys.2019.01298 PMC6798177

[15] H. Zhang , G. Qi , K. Wang , et al., “Oxidative Stress: Roles in Skeletal Muscle Atrophy,” Biochemical Pharmacology 214 (2023): 115664. 37331636 10.1016/j.bcp.2023.115664

[16] Y. Ji , J. Lin , R. Liu , et al., “Celecoxib Attenuates Hindlimb Unloading‐Induced Muscle Atrophy via Suppressing Inflammation, Oxidative Stress and ER Stress by Inhibiting STAT3,” Inflammopharmacology 32 (2024): 1633–1646. 38451396 10.1007/s10787-024-01454-7

[17] H. J. Zhang , B. H. Wang , X. Wang , et al., “Handelin Alleviates Cachexia‐ and Aging‐Induced Skeletal Muscle Atrophy by Improving Protein Homeostasis and Inhibiting Inflammation,” Journal of Cachexia, Sarcopenia and Muscle 15 (2024): 173–188. 38009816 10.1002/jcsm.13381 PMC10834327

[18] L. Zhang , M. Li , W. Wang , et al., “Celecoxib Alleviates Denervation‐Induced Muscle Atrophy by Suppressing Inflammation and Oxidative Stress and Improving Microcirculation,” Biochemical Pharmacology 203 (2022): 115186. 35882305 10.1016/j.bcp.2022.115186

[19] D. A. Gonçalves , W. A. Silveira , L. H. Manfredi , et al., “Insulin/IGF1 Signalling Mediates the Effects of β_2_‐Adrenergic Agonist on Muscle Proteostasis and Growth,” Journal of Cachexia, Sarcopenia and Muscle 10 (2019): 455–475. 30932373 10.1002/jcsm.12395 PMC6463755

[20] J. H. Park , J. Mok , S. Park , et al., “Celecoxib Enhances Oxidative Muscle Fibre Formation and Improves Muscle Functions Through Prokr1 Activation in Mice,” Journal of Cachexia, Sarcopenia and Muscle 16 (2025): e13704. 39887895 10.1002/jcsm.13704 PMC11780397

[21] H. Chen , Z. Qian , S. Zhang , et al., “Silencing COX‐2 Blocks PDK1/TRAF4‐Induced AKT Activation to Inhibit Fibrogenesis During Skeletal Muscle Atrophy,” Redox Biology 38 (2021): 101774. 33152664 10.1016/j.redox.2020.101774 PMC7645269

[22] M. Chang , R. Liu , B. Chen , et al., “hBMSC‐EVs Alleviate Weightlessness‐Induced Skeletal Muscle Atrophy by Suppressing Oxidative Stress and Inflammation,” Stem Cell Research & Therapy 16 (2025): 46. 39901193 10.1186/s13287-025-04175-y PMC11792267

[23] H. Liu , K. Wang , T. Shang , et al., “Astragaloside IV Improves Muscle Atrophy by Modulating the Activity of UPS and ALP via Suppressing Oxidative Stress and Inflammation in Denervated Mice,” Molecular Neurobiology 62 (2025): 4689–4704. 39480556 10.1007/s12035-024-04590-x

[24] M. J. Morgan and Y. S. Kim , “Roles of RIPK3 in Necroptosis, Cell Signaling, and Disease,” Experimental & Molecular Medicine 54 (2022): 1695–1704. 36224345 10.1038/s12276-022-00868-z PMC9636380

[25] H. T. Tran , T. Kratina , A. Coutansais , et al., “RIPK3 Cleavage Is Dispensable for Necroptosis Inhibition but Restricts NLRP3 Inflammasome Activation,” Cell Death and Differentiation 31 (2024): 662–671. 38514849 10.1038/s41418-024-01281-x PMC11094093

[26] W. Zhang , J. Zhang , Z. Wang , et al., “Extracellular RIPK3 Acts as a Damage‐Associated Molecular Pattern to Exaggerate Cardiac Ischemia/Reperfusion Injury,” Circulation 150 (2024): 1791–1811. 39411860 10.1161/CIRCULATIONAHA.123.068595

[27] J. S. Kang , N. J. Cho , S. W. Lee , et al., “RIPK3 Causes Mitochondrial Dysfunction and Albuminuria in Diabetic Podocytopathy Through PGAM5‐Drp1 Signaling,” Metabolism 159 (2024): 155982. 39089491 10.1016/j.metabol.2024.155982

[28] J. E. Morgan , A. Prola , V. Mariot , et al., “Necroptosis Mediates Myofibre Death in Dystrophin‐Deficient Mice,” Nature Communications 9 (2018): 3655. 10.1038/s41467-018-06057-9 PMC6128848 30194302

[29] M. Bencze , B. Periou , I. Punzón , et al., “Receptor Interacting Protein Kinase‐3 Mediates Both Myopathy and Cardiomyopathy in Preclinical Animal Models of Duchenne Muscular Dystrophy,” Journal of Cachexia, Sarcopenia and Muscle 14 (2023): 2520–2531. 37909859 10.1002/jcsm.13265 PMC10751447

[30] W. Ma , Y. Cai , Y. Shen , et al., “HDAC4 Knockdown Alleviates Denervation‐Induced Muscle Atrophy by Inhibiting Myogenin‐Dependent Atrogene Activation,” Frontiers in Cellular Neuroscience 15 (2021): 663384. 34276308 10.3389/fncel.2021.663384 PMC8278478

[31] Z. Huang , L. Zhong , J. Zhu , et al., “Inhibition of IL‐6/JAK/STAT3 Pathway Rescues Denervation‐Induced Skeletal Muscle Atrophy,” Annals Translated Medicine 8 (2020): 1681. 10.21037/atm-20-7269 PMC7812230 33490193

[32] S. E. Bollen , J. J. Bass , S. Fujita , D. Wilkinson , M. Hewison , and P. J. Atherton , “The Vitamin D/Vitamin D Receptor (VDR) Axis in Muscle Atrophy and Sarcopenia,” Cellular Signalling 96 (2022): 110355. 35595176 10.1016/j.cellsig.2022.110355

[33] S. Guan , L. Zhao , and R. Peng , “Mitochondrial Respiratory Chain Supercomplexes: From Structure to Function,” International Journal of Molecular Sciences 23, no. 22 (2022): 13880. 36430359 10.3390/ijms232213880 PMC9696846

[34] L. Chi , D. Lee , S. Leung , et al., “Loss of Functional Peroxisomes Leads to Increased Mitochondrial Biogenesis and Reduced Autophagy That Preserve Mitochondrial Function,” Cellular and Molecular Life Sciences 80 (2023): 183. 37338571 10.1007/s00018-023-04827-3 PMC10281899

[35] H. Sun , J. Sun , M. Li , et al., “Transcriptome Analysis of Immune Receptor Activation and Energy Metabolism Reduction as the Underlying Mechanisms in Interleukin‐6‐Induced Skeletal Muscle Atrophy,” Frontiers in Immunology 12 (2021): 730070. 34552592 10.3389/fimmu.2021.730070 PMC8450567

[36] L. Zanders , M. Kny , A. Hahn , et al., “Sepsis Induces Interleukin 6, gp130/JAK2/STAT3, and Muscle Wasting,” Journal of Cachexia, Sarcopenia and Muscle 13 (2022): 713–727. 34821076 10.1002/jcsm.12867 PMC8818599

[37] W. Y. Fang , Y. T. Tseng , T. Y. Lee , et al., “Triptolide Prevents LPS‐Induced Skeletal Muscle Atrophy via Inhibiting NF‐κB/TNF‐α and Regulating Protein Synthesis/Degradation Pathway,” British Journal of Pharmacology 178 (2021): 2998–3016. 33788266 10.1111/bph.15472

[38] Y. Lei , M. Gan , Y. Qiu , et al., “The Role of Mitochondrial Dynamics and Mitophagy in Skeletal Muscle Atrophy: From Molecular Mechanisms to Therapeutic Insights,” Cellular & Molecular Biology Letters 29 (2024): 59. 38654156 10.1186/s11658-024-00572-y PMC11036639

[39] A. Lozhkin , A. E. Vendrov , R. Ramos‐Mondragón , et al., “Mitochondrial Oxidative Stress Contributes to Diastolic Dysfunction Through Impaired Mitochondrial Dynamics,” Redox Biology 57 (2022): 102474. 36183542 10.1016/j.redox.2022.102474 PMC9530618

[40] C. Ma , K. Liu , F. Wang , et al., “Neutrophil Membrane‐Engineered Panax Ginseng Root‐Derived Exosomes Loaded miRNA 182‐5p Targets NOX4/Drp‐1/NLRP3 Signal Pathway to Alleviate Acute Lung Injury in Sepsis: Experimental Studies,” International Journal of Surgery 110 (2024): 72–86. 37737899 10.1097/JS9.0000000000000789 PMC10793765

