# Supplementary material for: RIPK3 Inhibition Mitigates Denervated Muscle Atrophy via NOX4‐Mediated Mitochondrial Restoration and Inflammation Suppression
Source: J Cachexia Sarcopenia Muscle. 2026 May 1;17(3):e70311. doi: 10.1002/jcsm.70311 (PMC13133598; doi:10.1002/jcsm.70311)
Supplement: Supplementary file 1 — Data S1: Supporting information. [file JCSM-17-e70311-s002.pdf]

Fig 1F

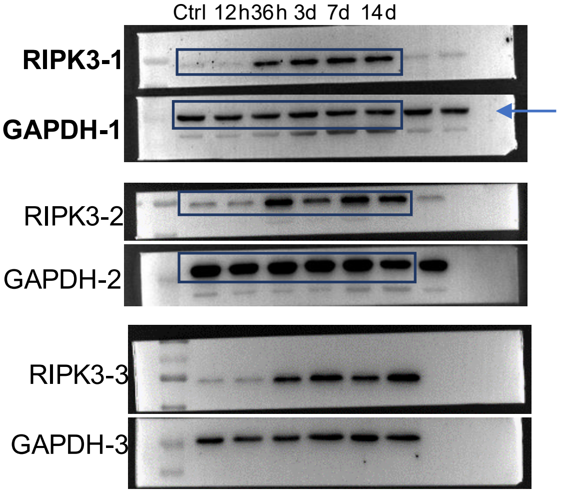

Fig 2F

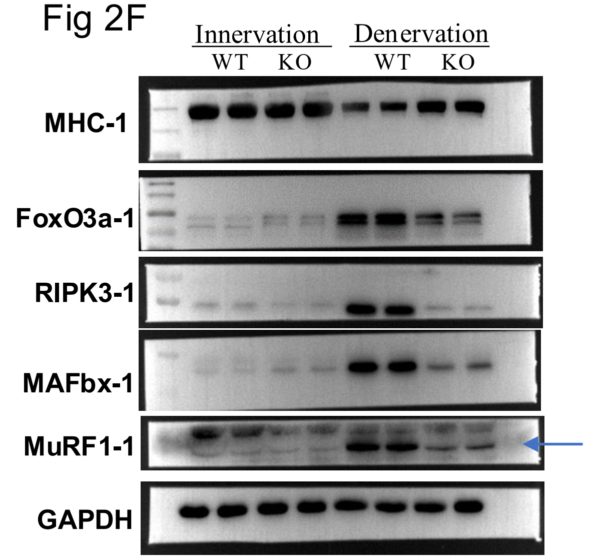

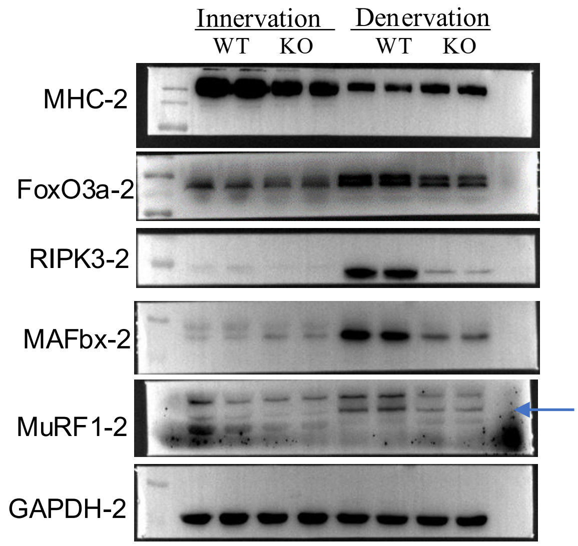

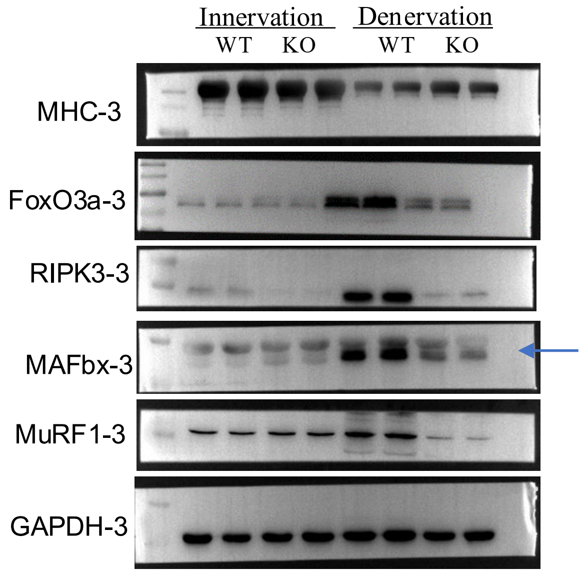

Fig 5B

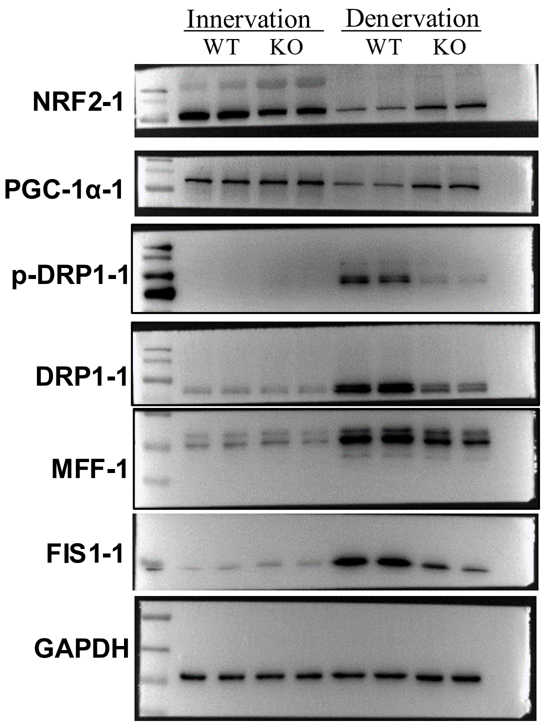

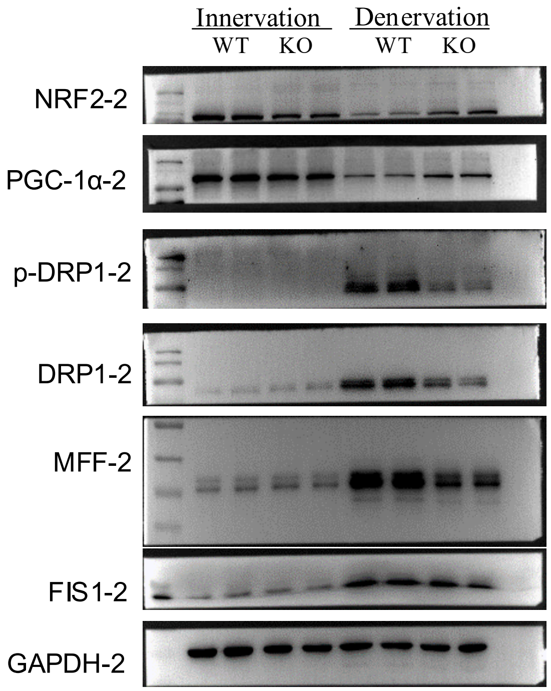

Fig 5B

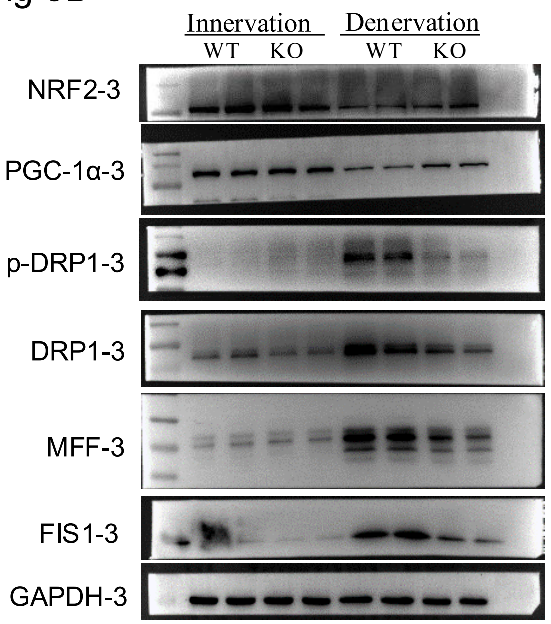

Fig 6B

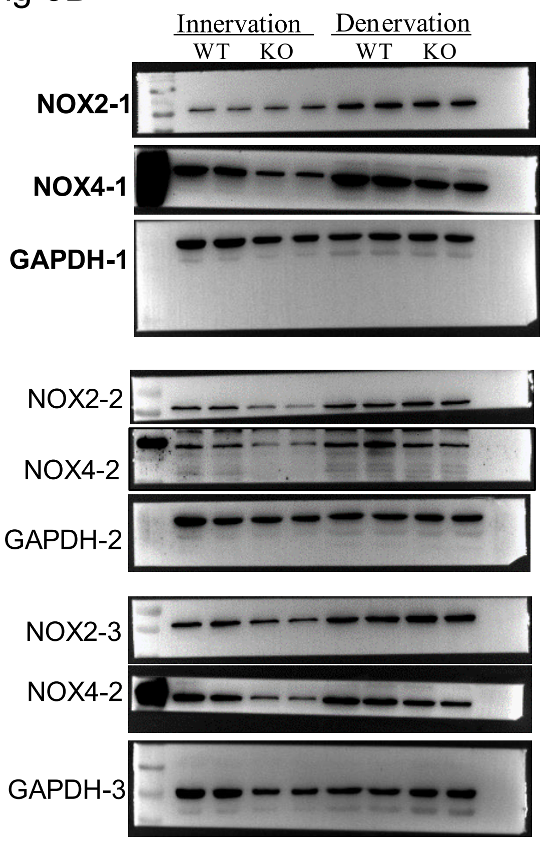

Fig 6F

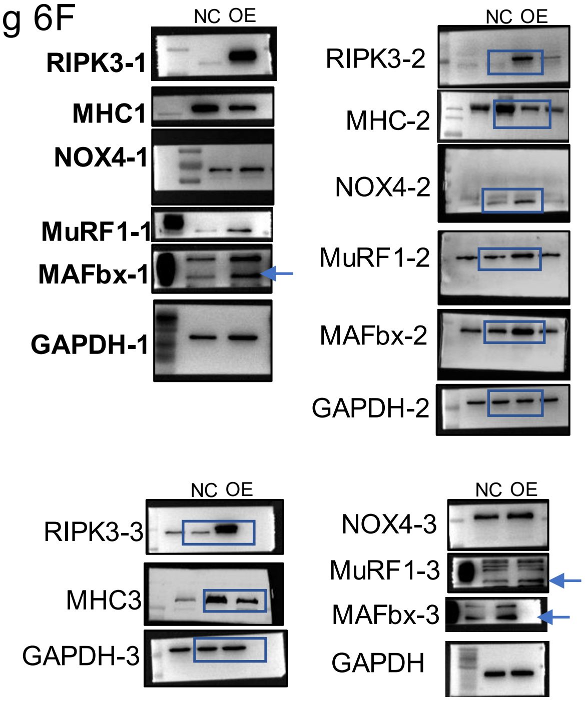

Fig 7F

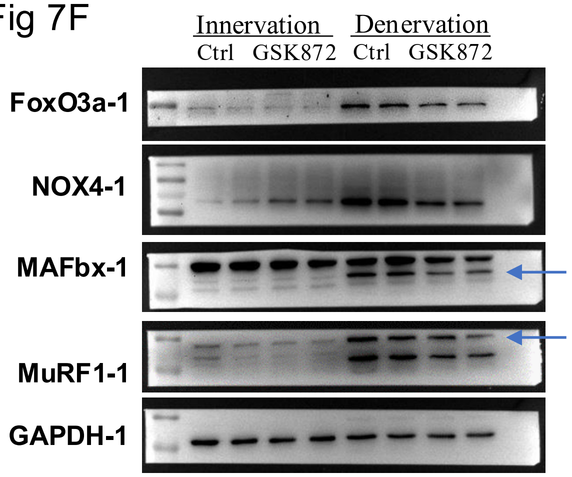

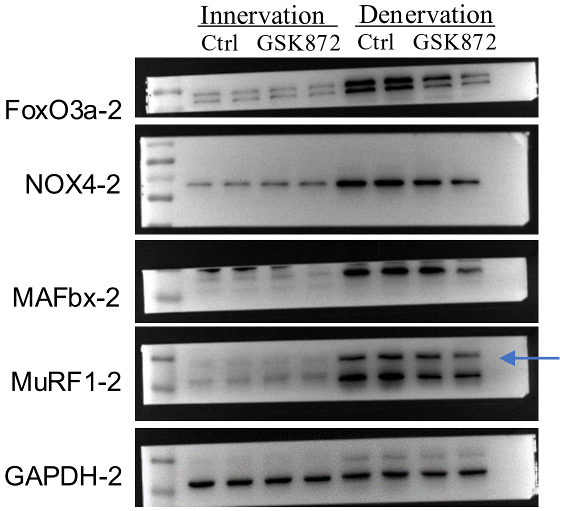

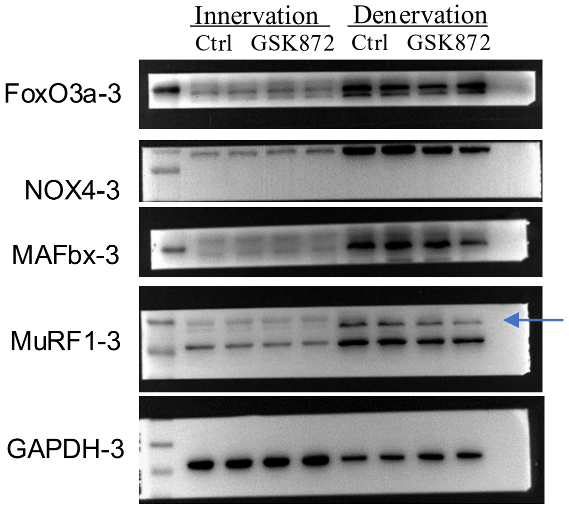

Fig 6D

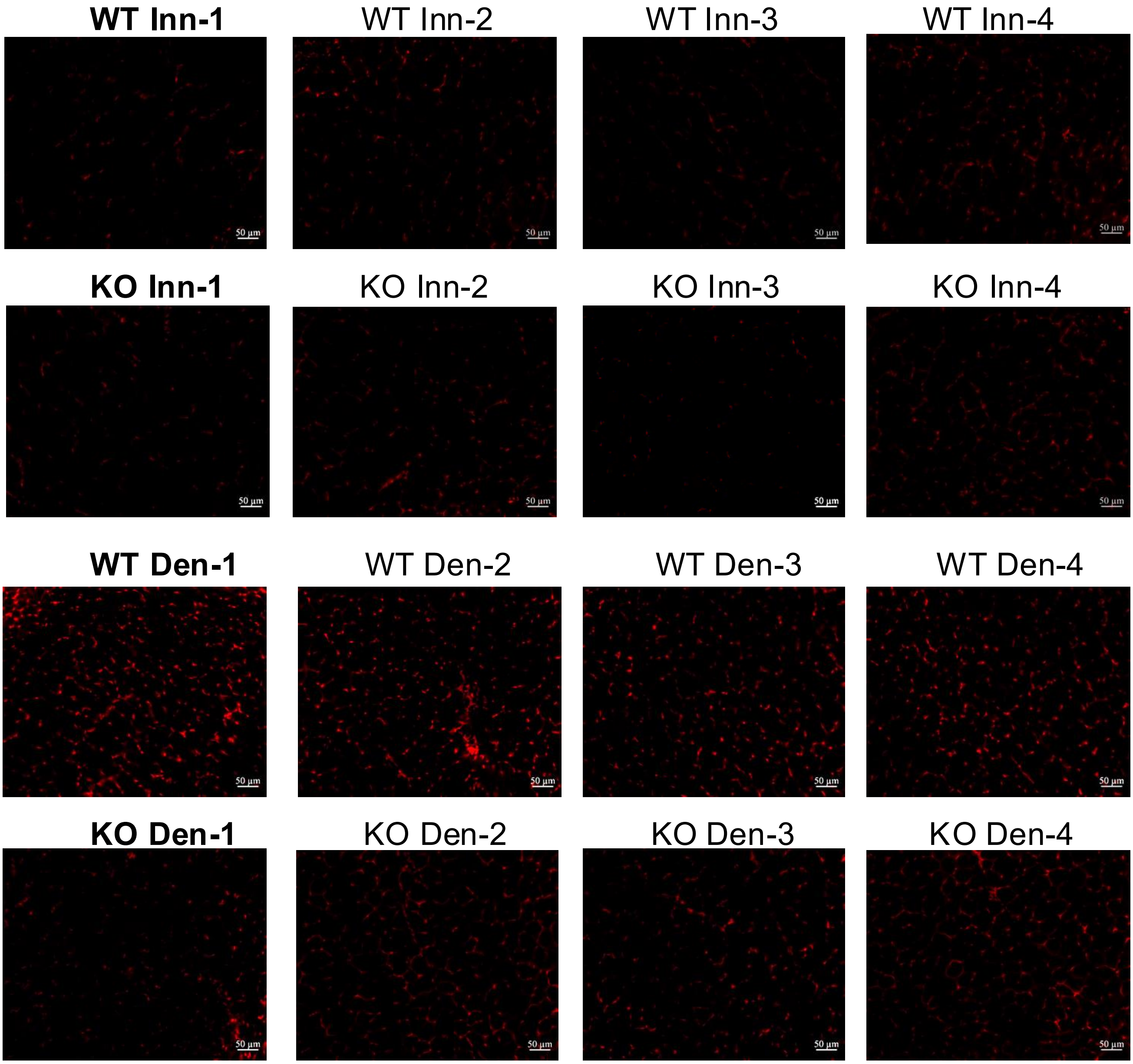
WT Inn-1
WT Inn-2
WT Inn-3
WT Inn-4
KO Inn-1
KO Inn-2
KO Inn-3
KO Inn-4
WT Den-1
WT Den-2
WT Den-3
WT Den-4
KO Den-1
KO Den-2
KO Den-3
KO Den-4
50 µm

Fig 5A

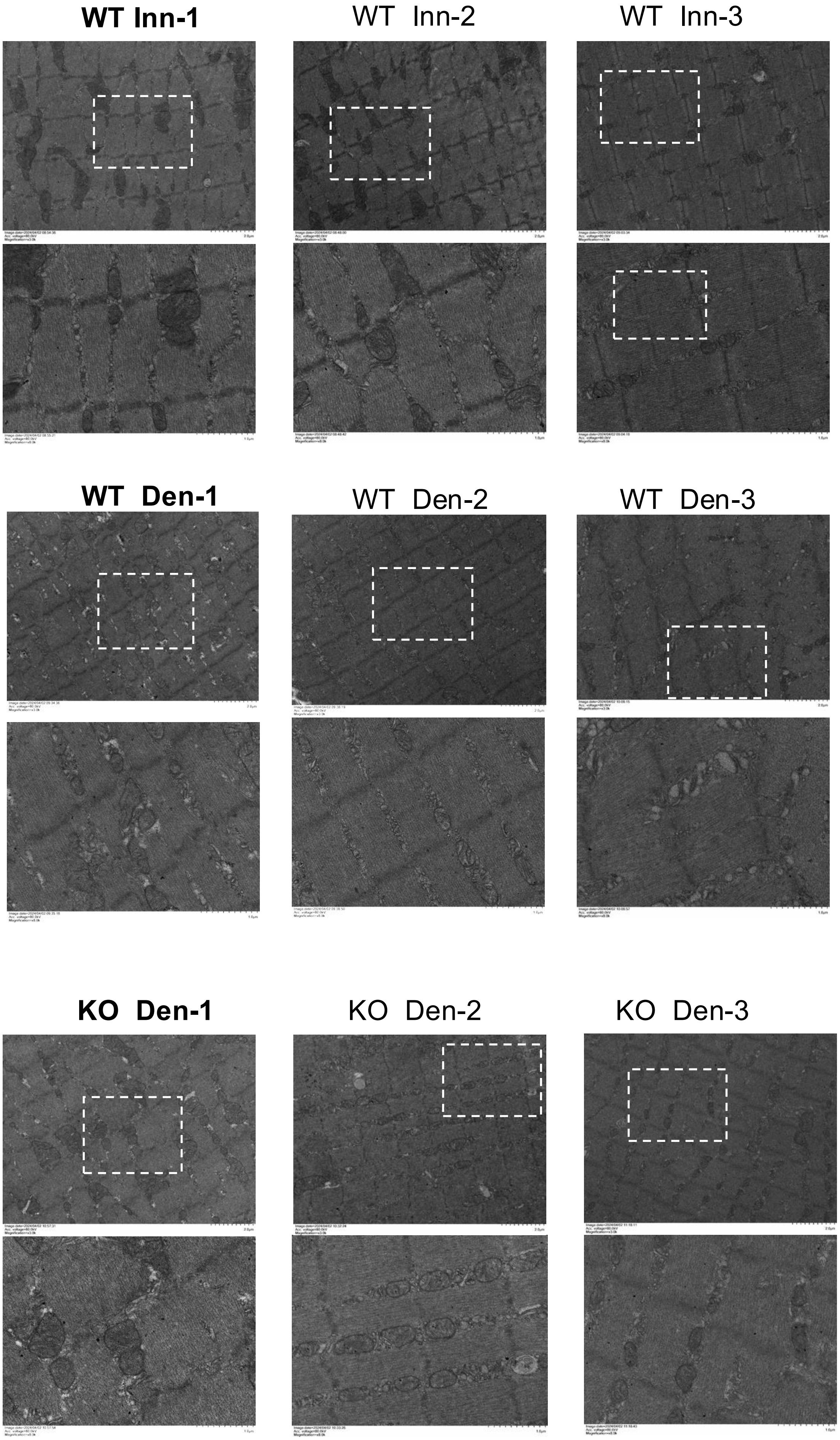
WT Inn-1
WT Inn-2
WT Inn-3
WT Den-1
WT Den-2
WT Den-3
KO Den-1
KO Den-2
KO Den-3
