# Supplementary material for: RIPK3 Inhibition Mitigates Denervated Muscle Atrophy via NOX4‐Mediated Mitochondrial Restoration and Inflammation Suppression
Source: J Cachexia Sarcopenia Muscle. 2026 May 1;17(3):e70311. doi: 10.1002/jcsm.70311 (PMC13133598; doi:10.1002/jcsm.70311)
Supplement: Supplementary file 2 — Table S1: Primer sequence for genes. Table S2: Statistical summary. [file JCSM-17-e70311-s001.pdf]

Supplementary Table 1. Primer sequence for genes.

Ndufb8	F	TGTTGCCGGGGTCATATCCTA
	R	AGCATCGGGTAGTCGCCATA
Sdhb	F	CAGAGTCGGCCTGCAGTTTC
	R	GGTCCCATCGGTAAATGGCA
Uqcrb	F	GGCCGATCTGCTGTTTCAG
	R	CATCTCGCATTAACCCCAGTT
Cox5a	F	GCCGCTGTCTGTTCCATTC
	R	GCATCAATGTCTGGCTTGTTGAA
Atp5e	F	CAGGCTGGACTCAGCTACATC
	R	GTTCGCTTTGAACTCGGTCTT
Nqo1	F	AGGATGGGAGGTACTCGAATC
	R	TGCTAGAGATGACTCGGAAGG
Sod1	F	AACCAGTTGTGTTGTCAGGAC
	R	CCACCATGTTTCTTAGAGTGAGG
Nox2	F	TGAATGCCAGAGTCGGGATTT
	R	CGAGTCACGGCCACATACA
Nox4	F	TGCCTGCTCATTTGGCTGT
	R	CCGGCACATAGGTAAAAGGATG
18S	F	CAGCCACCCGAGATTGAGCA
	R	TAGTAGCGACGGGCGGTGT

Supplementary Table 2. Statistical summary

Figure	Measurement	Groups (n)	Normality (Shapiro-Wilk)	Variance homogeneity (Brown-Forsythe)	Statistical test	Key results
Fig 1F	RIPK3 protein	Ctrl (3), 12h (3), 36h (3), 3d (3), 7d (3), 14d (3), 28d (3)	All groups $P > 0.05$	$P = 0.2555$ (homogeneous)	One-way ANOVA	$F_{5,12} = 49.04$, $P < 0.001$, $R^2 = 0.9533$<br>Post hoc: 36h–28d vs. Ctrl $P < 0.001$
Fig 2B	Wet weight ratio	TA-WT (12), TA-KO (12), GAS-WT (12), GAS-KO (12)	All groups $P > 0.05$	$P = 0.7693$ (homogeneous)	Two-way ANOVA (muscle × genotype)	Interaction: $F_{1,44} = 2.20$, $P = 0.1448$<br>Muscle: $F_{1,44} = 54.65$, $P < 0.001$<br>Genotype: $F_{1,44} = 9.81$, $P = 0.0031$<br>Post hoc: GAS-WT vs. GAS-KO $P = 0.0110$; TA-WT vs. TA-KO $P = 0.6520$
Fig 2D	GAS CSA	WT-Inn (7), KO-Inn (7), WT-Den (7), KO-Den (7)	All groups $P > 0.05$	$P = 0.5835$ (homogeneous)	Two-way ANOVA (surgery × genotype)	Interaction: $F_{1,24} = 0.45$, $P = 0.51$<br>Surgery: $F_{1,24} = 417.3$, $P < 0.001$<br>Genotype: $F_{1,24} = 11.51$, $P = 0.002$<br>Post hoc (Tukey):<br>• Inn-WT vs. Inn-KO: $P = 0.24$<br>• Inn-WT vs. Den-WT: $P < 0.001$<br>• Inn-KO vs. Den-KO: $P < 0.001$<br>• Den-WT vs. Den-KO: $P = 0.04$
Fig 2D	TA CSA	WT-Inn (7), KO-Inn (7), WT-Den (7), KO-Den (7)	KO-Den raw $P = 0.0347$ (non-normal); log-transformed $P = 0.0522$ (normal). All others $P > 0.05$.	Log-transformed data: $P = 0.9728$ (homogeneous)	Two-way ANOVA (surgery × genotype) on log-	Interaction: $F_{1,24} = 11.92$, $P = 0.0021$<br>Surgery: $F_{1,24} = 4.70$, $P = 0.0403$<br>Genotype: $F_{1,24} = 894.8$, $P < 0.001$<br>Post hoc (Tukey on log-transformed data):<br>• Inn-WT vs. Inn-KO: $P = 0.8008$

					transformed data	• Inn-WT vs. Den-WT: $P < 0.001$<br>• Inn-KO vs. Den-KO: $P < 0.001$<br>• Den-WT vs. Den-KO: $P = 0.0030$
Fig 2F	MHC protein	WT-Inn (3), KO-Inn (3), WT-Den (3), KO-Den (3)	All groups $P > 0.05$	$P = 0.6108$ (homogeneous)	Two-way ANOVA (surgery × genotype)	Interaction: $F_{1,8} = 12.84$, $P = 0.0072$<br>Surgery: $F_{1,8} = 2.38$, $P = 0.1616$<br>Genotype: $F_{1,8} = 106.5$, $P < 0.001$<br>Post hoc (Tukey):<br>• Inn-WT vs. Inn-KO: $P = 0.5097$<br>• Inn-WT vs. Den-WT: $P < 0.001$<br>• Inn-KO vs. Den-KO: $P = 0.0062$<br>• Den-WT vs. Den-KO: $P = 0.0278$
Fig 2F	FOXO3a protein	WT-Inn (3), KO-Inn (3), WT-Den (3), KO-Den (3)	All groups $P > 0.05$	$P = 0.9988$ (homogeneous)	Two-way ANOVA (surgery × genotype)	Interaction: $F_{1,8} = 22.98$, $P = 0.0014$<br>Surgery: $F_{1,8} = 15.75$, $P = 0.0041$<br>Genotype: $F_{1,8} = 112.4$, $P < 0.001$<br>Post hoc (Tukey):<br>• Inn-WT vs. Inn-KO: $P = 0.9343$<br>• Inn-WT vs. Den-WT: $P < 0.001$<br>• Inn-KO vs. Den-KO: $P = 0.0144$<br>• Den-WT vs. Den-KO: $P = 0.0012$
Fig 2F	MuRF1 protein	WT-Inn (3), KO-Inn (3), WT-Den (3), KO-Den (3)	All groups $P > 0.05$	$P = 0.3807$ (homogeneous)	Two-way ANOVA (surgery × genotype)	Interaction: $F_{1,8} = 13.00$, $P = 0.0069$<br>Surgery: $F_{1,8} = 15.92$, $P = 0.0040$<br>Genotype: $F_{1,8} = 39.67$, $P < 0.001$<br>Post hoc (Tukey):<br>• Inn-WT vs. Inn-KO: $P = 0.9924$<br>• Inn-WT vs. Den-WT: $P = 0.0005$

						• Inn-KO vs. Den-KO: $P = 0.2985$<br>• Den-WT vs. Den-KO: $P = 0.0030$
Fig 2F	MAFbx protein	WT-Inn (3), KO-Inn (3), WT-Den (3), KO-Den (3)	All groups $P > 0.05$	$P = 0.774$ (homogeneous)	Two-way ANOVA (surgery × genotype)	Interaction: $F_{1,8} = 25.49$, $P = 0.0010$<br>Surgery: $F_{1,8} = 21.56$, $P = 0.0017$<br>Genotype: $F_{1,8} = 81.20$, $P < 0.001$<br>Post hoc (Tukey):<br>• Inn-WT vs. Inn-KO: $P = 0.9911$<br>• Inn-WT vs. Den-WT: $P < 0.001$<br>• Inn-KO vs. Den-KO: $P = 0.0880$<br>• Den-WT vs. Den-KO: $P < 0.001$
Fig 4E	Ndufb8 mRNA	WT-Inn (4), KO-Inn (4), WT-Den (4), KO-Den (4)	All groups $P > 0.05$	$P = 0.6919$ (homogeneous)	Two-way ANOVA (surgery × genotype)	Interaction: $F_{1,12} = 14.0$, $P = 0.0028$<br>Surgery: $F_{1,12} = 21.8$, $P < 0.001$<br>Genotype: $F_{1,12} = 22.8$, $P < 0.001$<br>Post hoc (Tukey):<br>• Inn-WT vs. Inn-KO: $P = 0.8858$<br>• Inn-WT vs. Den-WT: $P < 0.001$<br>• Inn-KO vs. Den-KO: $P = 0.9137$<br>• Den-WT vs. Den-KO: $P < 0.001$
Fig 4E	Sdhb mRNA	WT-Inn (4), KO-Inn (4), WT-Den (4), KO-Den (4)	All groups $P > 0.05$	$P = 0.0973$ (homogeneous)	Two-way ANOVA (surgery × genotype)	Interaction: $F_{1,12} = 0.0017$, $P = 0.9677$<br>Surgery: $F_{1,12} = 5.83$, $P = 0.0326$<br>Genotype: $F_{1,12} = 87.3$, $P < 0.001$<br>Post hoc (Tukey):<br>• Inn-WT vs. Inn-KO: $P = 0.3756$<br>• Inn-WT vs. Den-WT: $P = 0.0001$<br>• Inn-KO vs. Den-KO: $P < 0.001$<br>• Den-WT vs. Den-KO: $P = 0.348$

Fig 4E	Uqcrb mRNA	WT-Inn (4), KO-Inn (4), WT-Den (4), KO-Den (4)	All groups $P > 0.05$	$P =$ 0.9486 (homogeneous)	Two-way ANOVA (surgery × genotype)	Interaction: $F_{1,12} = 0.280$, $P = 0.6063$<br>Surgery: $F_{1,12} = 13.2$, $P = 0.0035$<br>Genotype: $F_{1,12} = 3.69$, $P = 0.0787$<br>Post hoc (Tukey):<br>• Inn-WT vs. Inn-KO: $P = 0.1807$<br>• Inn-WT vs. Den-WT: $P = 0.3497$<br>• Inn-KO vs. Den-KO: $P = 0.7606$<br>• Den-WT vs. Den-KO: $P = 0.0525$
Fig 4E	Cox5a mRNA	WT-Inn (4), KO-Inn (4), WT-Den (4), KO-Den (4)	All groups $P > 0.05$	$P = 0.5433$ (homogeneous)	Two-way ANOVA (surgery × genotype)	Interaction: $F_{1,12} = 1.83$, $P = 0.2014$<br>Surgery: $F_{1,12} = 1.50$, $P = 0.2442$<br>Genotype: $F_{1,12} = 6.59$, $P = 0.0246$<br>Post hoc (Tukey):<br>• Inn-WT vs. Inn-KO: $P = 0.9997$<br>• Inn-WT vs. Den-WT: $P = 0.0701$<br>• Inn-KO vs. Den-KO: $P = 0.825$<br>• Den-WT vs. Den-KO: $P = 0.3105$
Fig 4E	Atp5e mRNA	WT-Inn (4), KO-Inn (4), WT-Den (4), KO-Den (4)	All groups $P > 0.05$	$P = 0.9657$ (homogeneous)	Two-way ANOVA (surgery × genotype)	Interaction: $F_{1,12} = 8.74$, $P = 0.0120$<br>Surgery: $F_{1,12} = 19.4$, $P < 0.001$<br>Genotype: $F_{1,12} = 23.2$, $P < 0.001$<br>Post hoc (Tukey):<br>• Inn-WT vs. Inn-KO: $P = 0.741$<br>• Inn-WT vs. Den-WT: $P < 0.001$<br>• Inn-KO vs. Den-KO: $P = 0.5712$<br>• Den-WT vs. Den-KO: $P = 0.0011$

Fig 4E	Nqo1 mRNA	WT-Inn (4), KO-Inn (4), WT-Den (4), KO-Den (4)	All groups $P > 0.05$	$P = 0.7369$ (homogeneous)	Two-way ANOVA (surgery × genotype)	Interaction: $F_{1,12} = 75.3$, $P < 0.001$<br>Surgery: $F_{1,12} = 24.6$, $P < 0.001$<br>Genotype: $F_{1,12} = 4.91$, $P = 0.0468$<br>Post hoc (Tukey):<br>• Inn-WT vs. Inn-KO: $P = 0.0895$<br>• Inn-WT vs. Den-WT: $P < 0.001$<br>• Inn-KO vs. Den-KO: $P = 0.0031$<br>• Den-WT vs. Den-KO: $P < 0.001$
Fig 4E	Sod1 mRNA	WT-Inn (4), KO-Inn (4), WT-Den (4), KO-Den (4)	All groups $P > 0.05$	$P = 0.1451$ (homogeneous)	Two-way ANOVA (surgery × genotype)	Interaction: $F_{1,12} = 21.90$, $P < 0.001$<br>Surgery: $F_{1,12} = 12.14$, $P = 0.0045$<br>Genotype: $F_{1,12} = 0.15$, $P = 0.7013$<br>Post hoc (Tukey):<br>• Inn-WT vs. Inn-KO: $P = 0.8319$<br>• Inn-WT vs. Den-WT: $P = 0.0170$<br>• Inn-KO vs. Den-KO: $P = 0.0449$<br>• Den-WT vs. Den-KO: $P < 0.001$
Fig 4F	Complex I activity	WT-Inn (3), KO-Inn (3), WT-Den (3), KO-Den (3)	All groups $P > 0.05$	$P = 0.4025$ (homogeneous)	Two-way ANOVA (surgery × genotype)	Interaction: $F_{1,8} = 6.22$, $P = 0.0373$<br>Surgery: $F_{1,8} = 158.8$, $P < 0.001$<br>Genotype: $F_{1,8} = 4.70$, $P = 0.0619$<br>Post hoc (Tukey):<br>• Inn-WT vs. Inn-KO: $P = 0.9954$<br>• Inn-WT vs. Den-WT: $P < 0.001$<br>• Inn-KO vs. Den-KO: $P < 0.001$<br>• Den-WT vs. Den-KO: $P = 0.0438$

Fig 4F	Complex V activity	WT-Inn (3), KO-Inn (3), WT-Den (3), KO-Den (3)	All groups $P > 0.05$	$P = 0.5191$ (homogeneous)	Two-way ANOVA (surgery × genotype)	Interaction: $F_{1,8} = 24.93$, $P = 0.0011$<br>Surgery: $F_{1,8} = 443.5$, $P < 0.001$<br>Genotype: $F_{1,8} = 16.81$, $P = 0.0034$<br>Post hoc (Tukey):<br>• Inn-WT vs. Inn-KO: $P = 0.919$<br>• Inn-WT vs. Den-WT: $P < 0.001$<br>• Inn-KO vs. Den-KO: $P < 0.001$<br>• Den-WT vs. Den-KO: $P < 0.001$
Fig 5B	NRF2 protein	WT-Inn (3), KO-Inn (3), WT-Den (3), KO-Den (3)	All groups $P > 0.05$	$P = 0.8302$ (homogeneous)	Two-way ANOVA (surgery × genotype)	Interaction: $F_{1,8} = 9.70$, $P = 0.0143$<br>Surgery: $F_{1,8} = 75.21$, $P < 0.001$<br>Genotype: $F_{1,8} = 6.70$, $P = 0.0322$<br>Post hoc (Tukey):<br>• Inn-WT vs. Inn-KO: $P = 0.9812$<br>• Inn-WT vs. Den-WT: $P < 0.001$<br>• Inn-KO vs. Den-KO: $P = 0.0183$<br>• Den-WT vs. Den-KO: $P = 0.0159$
Fig 5B	PGC-1α protein	WT-Inn (3), KO-Inn (3), WT-Den (3), KO-Den (3)	All groups $P > 0.05$	$P = 0.9425$ (homogeneous)	Two-way ANOVA (surgery × genotype)	Interaction: $F_{1,8} = 6.91$, $P = 0.0302$<br>Surgery: $F_{1,8} = 35.19$, $P < 0.001$<br>Genotype: $F_{1,8} = 10.89$, $P = 0.0109$<br>Post hoc (Tukey):<br>• Inn-WT vs. Inn-KO: $P = 0.9626$<br>• Inn-WT vs. Den-WT: $P = 0.0014$<br>• Inn-KO vs. Den-KO: $P = 0.1686$<br>• Den-WT vs. Den-KO: $P = 0.0129$

Fig 5B	p-DRP1 protein	WT-Inn (3), KO-Inn (3), WT-Den (3), KO-Den (3)	All groups $P > 0.05$	$P = 0.4026$ (homogeneous)	Two-way ANOVA (surgery × genotype)	Interaction: $F_{1,8} = 18.25$, $P = 0.0027$<br>Surgery: $F_{1,8} = 62.98$, $P < 0.001$<br>Genotype: $F_{1,8} = 16.26$, $P = 0.0038$<br>Post hoc (Tukey):<br>• Inn-WT vs. Inn-KO: $P = 0.9981$<br>• Inn-WT vs. Den-WT: $P < 0.001$<br>• Inn-KO vs. Den-KO: $P = 0.1184$<br>• Den-WT vs. Den-KO: $P = 0.0017$
Fig 5B	DRP1 protein	WT-Inn (3), KO-Inn (3), WT-Den (3), KO-Den (3)	All groups $P > 0.05$	$P = 0.2506$ (homogeneous)	Two-way ANOVA (surgery × genotype)	Interaction: $F_{1,8} = 7.49$, $P = 0.0256$<br>Surgery: $F_{1,8} = 20.13$, $P = 0.0020$<br>Genotype: $F_{1,8} = 8.38$, $P = 0.0200$<br>Post hoc (Tukey):<br>• Inn-WT vs. Inn-KO: $P = 0.9994$<br>• Inn-WT vs. Den-WT: $P = 0.0041$<br>• Inn-KO vs. Den-KO: $P = 0.6225$<br>• Den-WT vs. Den-KO: $P = 0.0171$
Fig 5B	FIS1 protein	WT-Inn (3), KO-Inn (3), WT-Den (3), KO-Den (3)	All groups $P > 0.05$	$P = 0.5474$ (homogeneous)	Two-way ANOVA (surgery × genotype)	Interaction: $F_{1,8} = 67.09$, $P < 0.001$<br>Surgery: $F_{1,8} = 233.2$, $P < 0.001$<br>Genotype: $F_{1,8} = 61.57$, $P < 0.001$<br>Post hoc (Tukey):<br>• Inn-WT vs. Inn-KO: $P = 0.9945$<br>• Inn-WT vs. Den-WT: $P < 0.001$<br>• Inn-KO vs. Den-KO: $P = 0.0046$<br>• Den-WT vs. Den-KO: $P < 0.001$

Fig 5B	MFF protein	WT-Inn (3), KO-Inn (3), WT-Den (3), KO-Den (3)	All groups $P > 0.05$	$P = 0.1145$ (homogeneous)	Two-way ANOVA (surgery × genotype)	Interaction: $F_{1,8} = 13.94$, $P = 0.0058$<br>Surgery: $F_{1,8} = 41.71$, $P < 0.001$<br>Genotype: $F_{1,8} = 12.92$, $P = 0.0070$<br>Post hoc (Tukey):<br>• Inn-WT vs. Inn-KO: $P = 0.9996$<br>• Inn-WT vs. Den-WT: $P < 0.001$<br>• Inn-KO vs. Den-KO: $P = 0.2902$<br>• Den-WT vs. Den-KO: $P = 0.0037$
Fig 6B	NOX2 mRNA	WT-Inn (4), KO-Inn (4), WT-Den (4), KO-Den (4)	All groups $P > 0.05$	$P = 0.5581$ (homogeneous)	Two-way ANOVA (surgery × genotype)	Interaction: $F_{1,12} = 10.5$, $P = 0.0070$<br>Surgery: $F_{1,12} = 25.8$, $P < 0.001$<br>Genotype: $F_{1,12} = 7.03$, $P = 0.0212$<br>Post hoc (Tukey):<br>• Inn-WT vs. Inn-KO: $P = 0.9737$<br>• Inn-WT vs. Den-WT: $P < 0.001$<br>• Inn-KO vs. Den-KO: $P = 0.5844$<br>• Den-WT vs. Den-KO: $P = 0.0062$
Fig 6B	NOX4 mRNA	WT-Inn (4), KO-Inn (4), WT-Den (4), KO-Den (4)	All groups $P > 0.05$	$P = 0.1208$ (homogeneous)	Two-way ANOVA (surgery × genotype)	Interaction: $F_{1,12} = 5.76$, $P = 0.0336$<br>Surgery: $F_{1,12} = 70.4$, $P < 0.001$<br>Genotype: $F_{1,12} = 9.16$, $P = 0.0105$<br>Post hoc (Tukey):<br>• Inn-WT vs. Inn-KO: $P = 0.9695$<br>• Inn-WT vs. Den-WT: $P < 0.001$<br>• Inn-KO vs. Den-KO: $P = 0.0055$<br>• Den-WT vs. Den-KO: $P = 0.011$

Fig 6B	NOX2 protein	WT-Inn (3), KO-Inn (3), WT-Den (3), KO-Den (3)	All groups $P > 0.05$	$P = 0.9627$ (homogeneous)	Two-way ANOVA (surgery × genotype)	Interaction: $F_{1,8} = 0.117$, $P = 0.7408$<br>Surgery: $F_{1,8} = 209.2$, $P < 0.001$<br>Genotype: $F_{1,8} = 0.217$, $P = 0.6538$<br>Post hoc (Tukey):<br>• Inn-WT vs. Inn-KO: $P = 0.9997$<br>• Inn-WT vs. Den-WT: $P < 0.001$<br>• Inn-KO vs. Den-KO: $P < 0.001$<br>• Den-WT vs. Den-KO: $P = 0.9378$
Fig 6B	NOX4 protein	WT-Inn (3), KO-Inn (3), WT-Den (3), KO-Den (3)	All groups $P > 0.05$	$P > 0.9999$ (homogeneous)	Two-way ANOVA (surgery × genotype)	Interaction: $F_{1,8} = 3.22$, $P = 0.1105$<br>Surgery: $F_{1,8} = 8.46$, $P = 0.0197$<br>Genotype: $F_{1,8} = 9.30$, $P = 0.0158$<br>Post hoc (Tukey):<br>• Inn-WT vs. Inn-KO: $P = 0.8117$<br>• Inn-WT vs. Den-WT: $P = 0.0421$<br>• Inn-KO vs. Den-KO: $P = 0.8584$<br>• Den-WT vs. Den-KO: $P = 0.0366$
Fig 6D	DHE staining (ROS)	WT-Inn (4), KO-Inn (4), WT-Den (4), KO-Den (4)	All groups $P > 0.05$	Log-transformed data: $P = 0.7211$ (homogeneous)	Two-way ANOVA (surgery × genotype) on log-transformed data	Interaction: $F_{1,12} = 43.4$, $P < 0.001$<br>Surgery: $F_{1,12} = 1122$, $P < 0.001$<br>Genotype: $F_{1,12} = 43.0$, $P < 0.001$<br>Post hoc (Tukey on log-transformed data):<br>• Inn-WT vs. Inn-KO: $P > 0.9999$<br>• Inn-WT vs. Den-WT: $P < 0.001$<br>• Inn-KO vs. Den-KO: $P < 0.001$<br>• Den-WT vs. Den-KO: $P < 0.001$

Fig 6F	C2C12 myotube protein levels	MHC-NC (3), MHC-OE (3), MuRF1-NC (3), MuRF1-OE (3), MAFbx-NC (3), MAFbx-OE (3), NOX4-NC (3), NOX4-OE (3)	All groups $P > 0.05$	$P = 0.8907$ (homogeneous)	Two-way ANOVA (protein × treatment)	Interaction: $F_{3,16} = 21.78$, $P < 0.001$<br>Protein: $F_{3,16} = 21.98$, $P < 0.001$<br>Treatment: $F_{1,16} = 37.33$, $P < 0.001$<br>Post hoc (Tukey, NC vs. OE for each protein):<br>• MHC: $P = 0.0307$<br>• MuRF1: $P < 0.001$<br>• MAFbx: $P = 0.0097$<br>• NOX4: $P = 0.0045$
Fig 7B	Wet weight ratio (TA and GAS)	TA-Ctrl (12), TA-GSK872 (12), GAS-Ctrl (12), GAS-GSK872 (12)	All groups $P > 0.05$	$P = 0.7389$ (homogeneous)	Two-way ANOVA (muscle × treatment)	Interaction: $F_{1,44} = 0.023$, $P = 0.8803$<br>Muscle: $F_{1,44} = 234.9$, $P < 0.001$<br>Treatment: $F_{1,44} = 15.75$, $P = 0.0003$<br>Post hoc (Tukey):<br>• TA Ctrl vs. TA GSK872: $P = 0.0467$<br>• GAS Ctrl vs. GAS GSK872: $P = 0.0277$
Fig 7C–D	GAS CSA	Inn-Ctrl (7), Inn-GSK872 (7), Den-Ctrl (7), Den-GSK872 (7)	All groups $P > 0.05$	$P = 0.2101$ (homogeneous)	Two-way ANOVA (surgery × treatment)	Interaction: $F_{1,24} = 1.93$, $P = 0.1778$<br>Surgery: $F_{1,24} = 706.2$, $P < 0.001$<br>Treatment: $F_{1,24} = 6.46$, $P = 0.0179$<br>Post hoc (Tukey):<br>• Inn Ctrl vs. Inn GSK872: $P = 0.8463$<br>• Inn Ctrl vs. Den GSK872: $P < 0.001$<br>• Inn GSK872 vs. Den GSK872: $P < 0.001$<br>• Den Ctrl vs. Den GSK872: $P = 0.0478$
Fig 7E	FOXO3a protein	Inn-Ctrl (3), Inn-GSK872 (3), Den-Ctrl (3), Den-GSK872 (3)	All groups $P > 0.05$	$P = 0.3711$ (homogeneous)	Two-way ANOVA (surgery × treatment)	Interaction: $F_{1,8} = 7.16$, $P = 0.0281$<br>Surgery: $F_{1,8} = 16.91$, $P = 0.0034$<br>Treatment: $F_{1,8} = 4.95$, $P = 0.0568$<br>Post hoc (Tukey):

						• Inn Ctrl vs. Inn GSK872: $P = 0.9879$<br>• Inn Ctrl vs. Den GSK872: $P = 0.0059$<br>• Inn GSK872 vs. Den GSK872: $P = 0.7453$<br>• Den Ctrl vs. Den GSK872: $P = 0.0347$
Fig 7E	MuRF1 protein	Inn-Ctrl (3), Inn-GSK872 (3), Den-Ctrl (3), Den-GSK872 (3)	All groups $P > 0.05$	$P = 0.9114$ (homogeneous)	Two-way ANOVA (surgery × treatment)	Interaction: $F_{1,8} = 11.96$, $P = 0.0086$<br>Surgery: $F_{1,8} = 235.4$, $P < 0.001$<br>Treatment: $F_{1,8} = 12.90$, $P = 0.0071$<br>Post hoc (Tukey):<br>• Inn Ctrl vs. Inn GSK872: $P = 0.9997$<br>• Inn Ctrl vs. Den GSK872: $P < 0.001$<br>• Inn GSK872 vs. Den GSK872: $P < 0.001$<br>• Den Ctrl vs. Den GSK872: $P = 0.0047$
Fig 7E	MAFbx protein	Inn-Ctrl (3), Inn-GSK872 (3), Den-Ctrl (3), Den-GSK872 (3)	All groups $P > 0.05$	$P = 0.7564$ (homogeneous)	Two-way ANOVA (surgery × treatment)	Interaction: $F_{1,8} = 9.02$, $P = 0.0170$<br>Surgery: $F_{1,8} = 40.75$, $P = 0.0002$<br>Treatment: $F_{1,8} = 10.57$, $P = 0.0117$<br>Post hoc (Tukey):<br>• Inn Ctrl vs. Inn GSK872: $P = 0.9979$<br>• Inn Ctrl vs. Den GSK872: $P < 0.001$<br>• Inn GSK872 vs. Den GSK872: $P = 0.1566$<br>• Den Ctrl vs. Den GSK872: $P = 0.0095$
Fig 7E	NOX4 protein	Inn-Ctrl (3), Inn-GSK872 (3), Den-Ctrl (3), Den-GSK872 (3)	All groups $P > 0.05$	$P = 0.7708$ (homogeneous)	Two-way ANOVA (surgery × treatment)	Interaction: $F_{1,8} = 10.79$, $P = 0.0111$<br>Surgery: $F_{1,8} = 109.0$, $P < 0.001$<br>Treatment: $F_{1,8} = 2.17$, $P = 0.1788$<br>Post hoc (Tukey):<br>• Inn Ctrl vs. Inn GSK872: $P = 0.5987$<br>• Inn Ctrl vs. Den GSK872: $P < 0.001$

	• Inn GSK872 vs. Den GSK872: $P = 0.0043$<br>• Den Ctrl vs. Den GSK872: $P = 0.0398$
